# Synthesis, DNA Binding and Antitumor Evaluation of Styelsamine and Cystodytin Analogues

**DOI:** 10.3390/md11020274

**Published:** 2013-01-28

**Authors:** Hugo K. H. Fong, Brent R. Copp

**Affiliations:** School of Chemical Sciences, University of Auckland, Private Bag 92019, Auckland, New Zealand; E-Mail: hfon009@aucklanduni.ac.nz

**Keywords:** marine natural products, styelsamine, cystodytin, pyridoacridine, DNA binding

## Abstract

A series of N-14 sidechain substituted analogues of styelsamine (pyrido[4,3,2-*mn*]acridine) and cystodytin (pyrido[4,3,2-*mn*]acridin-4-one) alkaloids have been prepared and evaluated for their DNA binding affinity and antiproliferative activity towards a panel of human tumor cell lines. Overall it was found that styelsamine analogues were stronger DNA binders, with the natural products styelsamines B and D having particularly high affinity (*K*_app_ 5.33 × 10^6^ and 3.64 × 10^6^ M^−1^, respectively). In comparison, the cystodytin iminoquinone alkaloids showed lower affinity for DNA, but were typically just as active as styelsamine analogues at inhibiting proliferation of tumor cells *in vitro*. Sub-panel selectivity towards non-small cell lung, melanoma and renal cancer cell lines were observed for a number of the analogues. Correlation was observed between whole cell activity and clogP, with the most potent antiproliferative activity being observed for 3-phenylpropanamide analogues **37** and **41** (NCI panel average GI_50_ 0.4 μM and 0.32 μM, respectively) with clogP ~4.0–4.5.

## 1. Introduction

**Figure 1:**
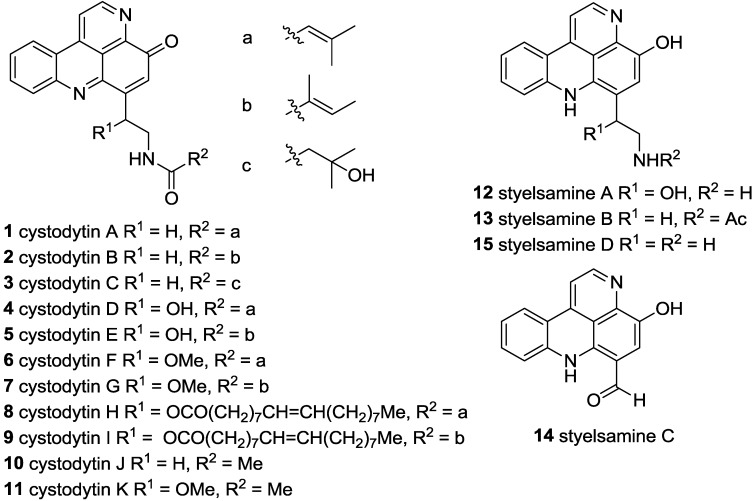
Structures of cystodytin and styelsamine natural products.

A diverse array of bioactive alkaloids isolated from marine sources contain the pyrido[4,3,2-*mn*]acridine scaffold [1]. While more structurally complex congeners are known, the simplest examples of such alkaloids are the tetracyclic cystodytins A–K (**1**–**11**) and styelsamines A–D (**12**–**15**) (Figure 1). The cystodytins, isolated from ascidians *Cystodytes dellechiajei* (**1**–**9**) [2,3], *Cystodytes* sp. (**10**) [4] and *Lissoclinum notti* (**11**) [5] possess the alkaloidal skeleton in the iminoquinone oxidation state, with modifications at either C-12 or N-14 of the ethylamine sidechain. Cytotoxicity towards murine or human tumor cell lines has been reported for the family of alkaloids, with IC_50_ values of 0.6 µM (L12010 murine lymphoma, **1**/**2**), 0.6 µM (L1210, **3**), 2.9 µM (L1210, **4**/**5**), 0.18 µM (L1210, **6**/**7**), 0.12 µM (L1210, **8**/**9**), 1.6 µM (HCT-116 human colon, **10**), and 1.3 µM (P388 murine leukemia, **11**) suggesting some influence of *C*-12 substitution on potency. Styelsamines A–D (**12**–**15**) were isolated as cytotoxic constituents of the Indonesian ascidian *Eusynstyela latericius* [6]. Moderate cytotoxicity towards the HCT-116 human colon tumor cell line with IC_50_ values of 33, 89, 2.6 and 1.6 µM were observed for each of **12**–**15** respectively. Styelsamine D is considered to play a central role in the biogenesis of many pyridoacridine alkaloids [7], though no definitive biosynthetic studies have been reported to date [8].

Pyridoacridine and pyridoacridine alkaloids typically exhibit wide-ranging biological properties including cytotoxicity, antibacterial and antiviral activities [9]. While it is often speculated that the bioactivity of pyridoacridine alkaloids is attributable to DNA binding [9], it has been noted by others that such a correlation is compound specific [4]. In the specific case of the cystodytins and styelsamines, all of the natural products have been evaluated for cytotoxicity, exhibiting a range of potency (IC_50_ 0.12–2.9 μM) [2,3,4,5] but only the DNA binding ability of cystodytin J (**10**) has been reported (*K*_disp_ 54 μM) [4]. As the natural products have only been evaluated against a limited range of tumor cell lines (e.g., murine lymphoma, murine leukemia and human colon) information is lacking as to the presence or not of any cell line selectivity for pyridoacridine alkaloids.

In an effort to explore the influence of N-14 substitution on the observed biological activities of styelsamine and cystodytin alkaloids, we have prepared a library of natural and un-natural analogues and evaluated their DNA affinity, using an ethidium bromide displacement assay, and cytotoxicity towards a panel of human tumor cell lines.

## 2. Results and Discussion

### 2.1. Chemistry

**Scheme 1:**
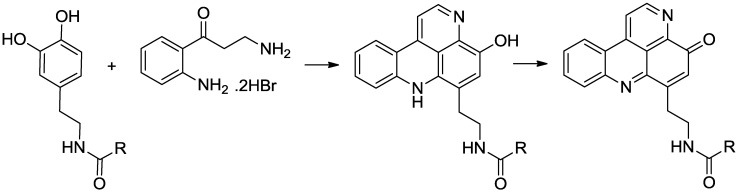
General reaction sequence for the preparation of styelsamine and cystodytin analogues.

The overall reaction sequence used to synthesize the target compounds is summarized in Scheme 1. This biomimetic method, first reported by Skyler and Heathcock [10] in their synthesis of styelsamine B, utilizes oxidative coupling of functionalized dopamine analogues with kynuramine to yield the desired pyridoacridine and pyridoacridone skeletons.

**Scheme 2:**
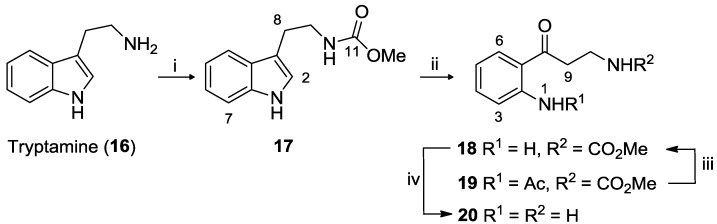
Preparation of kynuramine dihydrobromide **20**. *Reagents and conditions*: (i) methyl chloroformate, EtOAc/NaOH (1:0.6), N_2_, RT, 30 min, 73%; (ii) O_3_, AcOH, 0 °C, then conc. HCl, N_2_, 40 °C, 1.5 h, 42% (**18**) and 10% (**19**); (iii) aq. HCl, reflux, 4 h, 66% over two steps; (iv) HBr sat. AcOH, N_2_, 80 °C, 18 h, 96%.

Kynuramine dihydrobromide was prepared using a slightly modified version of the procedure previously reported [10]. Tryptamine (**16**) was first protected by conversion to the methyl carbamate **17**, achieved in 73% yield (Scheme 2). 

Whereas ozonolysis (in glacial acetic acid) of **17** has been previously reported to yield exclusively the keto-aniline **18**, in our hands we also observed the presence of a minor product (10% yield), determined to be the acetamide **19**. Hydrolysis of the crude reaction product containing both **18** and **19** in 10% aq. HCl afforded **18** (66% yield over two steps) while subsequent reaction with HBr in AcOH cleaved the carbamate protecting group to afford kynuramine dihydrobromide (**20**) in 96% yield. 

**Scheme 3:**
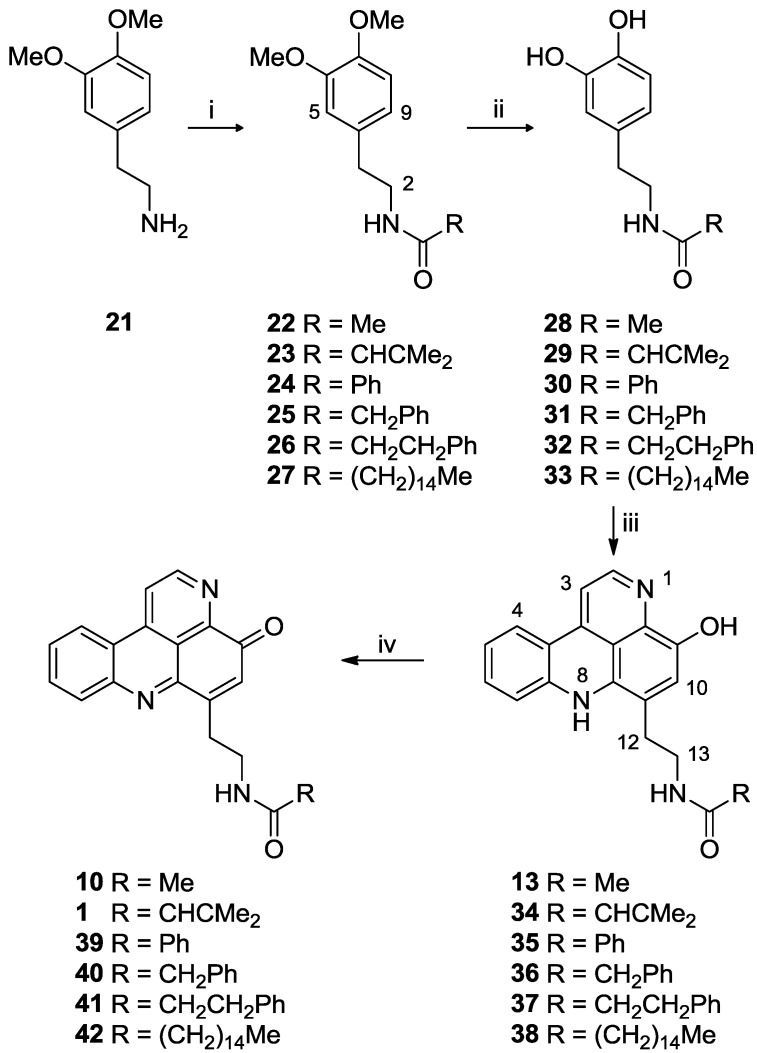
Synthesis of styelsamine (**13**, **34**–**38**) and cystodytin (**1**, **10**, **39**–**42**) analogues. *Reagents and conditions*: (i) for **22**: Ac_2_O, N_2_, RT, 1 h, 95%; for **23** and **25**: carboxylic acid, DMF, PyBOP, Et_3_N, RT, 18 h, 92% (**23**) and 99% (**25**); for **24**, **26** and **27**: acyl chloride, THF, Et_3_N, 0 °C rising to RT, 57% (**24**), 93% (**26**), 65% (**27**); (ii) BBr_3_, CH_2_Cl_2_, N_2_, −20 °C, 20 h, 90% (**28**), 98% (**29**), 85% (**30**), 79% (**31**), 89% (**32**), 75% (**33**); (iii) kynuramine dihydrobromide (**20**), CeCl_3_.7H_2_O, Ag_2_O, MeOH/AcOH (2:1), 19% (**13**), 6% (**34**), 15% (**35**), 11% (**36**), 20% (**37**), 16% (**38**); (iv) Ag_2_O, MeOH, 79% (**10**), 62% (**1**), 52% (**39**), 13% (**40**), 71% (**41**), 17% (**42**).

The requisite *N*-acyl dopamine analogues were prepared in two-step sequences from 3,4-dimethoxyphenethylamine (**21**) via one of three routes (Scheme 3). Thus acetamide **22** was synthesized in 95% yield by reaction of **21** with acetic anhydride, amides **23** (92%) and **25** (99%) were prepared by reaction of **21** with the appropriate carboxylic acid using PyBOP as a coupling agent in DMF, while amides **24**, **26** and **27** were prepared in yields of 57%, 93% and 65% respectively by reaction of amine **21** with the appropriate acid chloride in THF with Et_3_N. Demethylation of **22**–**27** by reaction with BBr_3_ (10 equiv.) in dry CH_2_Cl_2_ for 19 h gave the desired *N*-acyl dopamine analogues **28**–**33** in yields of 90%, 98%, 85%, 79%, 89%, and 75%, respectively.

Using the general methodology reported by Skyler and Heathcock [10], reaction of the *N*-acyldopamine analogues with kynuramine dihydrobromide (1.05 mole equiv.) in MeOH/AcOH (2:1) to which were added CeCl_3_·7H_2_O (0.15 mole equiv.) and silver (I) oxide (2 mole equiv.), afforded, after chromatographic purification, styelsamine B (**13**) and analogues **34**–**38** in yields of 19%, 6%, 15%, 11%, 20% and 16% respectively (Scheme 3). In each case, high resolution ESI-MS gave a pseudomolecular ion consistent with the presence of the expected product of the reaction. The ^1^H and ^13^C NMR spectra of **13**, **35**–**38** all contained the resonances expected for the styelsamine NH-1 to CH_2_-13 scaffold, with anticipated variation in NH-14 amide substitution. Data observed for **13** were in agreement with literature [6].

Each of the pyridoacridine alkaloids **13**, **34**–**38** were then oxidized to the corresponding pyridoacridine analogue by reaction with Ag_2_O (1 equiv.) in MeOH with NaHCO_3_ (Scheme 3). The purple coloration of the aminophenol starting materials was observed to quickly convert (2 min) to the yellow color of the iminoquinone chromophore. After workup, iminoquinones **10**, **1**, **39**–**42** were obtained in yields of 79%, 62%, 52%, 13%, 71%, and 17%, respectively.

For each of these products, ESI-MS identified a pseudomolecular ion two mass units lower than that observed for the corresponding pyridoacridine precursor. While complete NMR characterization of the reaction products was problematic due to their reduced solubility, evidence of successful formation of the iminoquinone scaffold was evidenced by changes in chemical shift of H-10. In the case of the aminophenol styelsamines, H-10 is observed between δ_H_ 7.14 and 7.51, while for the iminoquinone cystodytins, H-10 resonates between δ_H_ 6.81 and 7.01. In the specific cases of **1** (cystodytin A) [2] and **10** (cystodytin J) [4], MS and NMR data agreed with those reported for the natural products.

**Scheme 4:**
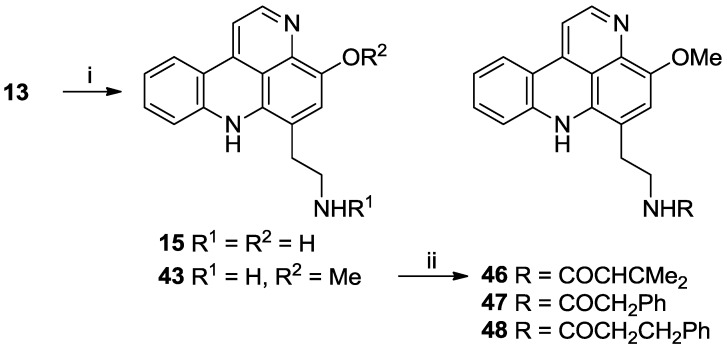
Synthesis of *O*-methylstyelsamine D (**43**) and amide analogues **46**–**48**. *Reagents and conditions*: (i) MeOH/4N HCl (1:1), 80 °C, 48 h, 60% (**15**) and 45% (**43**); (ii) for **46** and **47**: RCO_2_H, CH_2_Cl_2_, Et_3_N, PyBOP, 88% (**46**), 48% (**47**); for **48**: dihydrocinnamoylchloride, THF, Et_3_N, 30 min, 43%.

**Figure 2:**
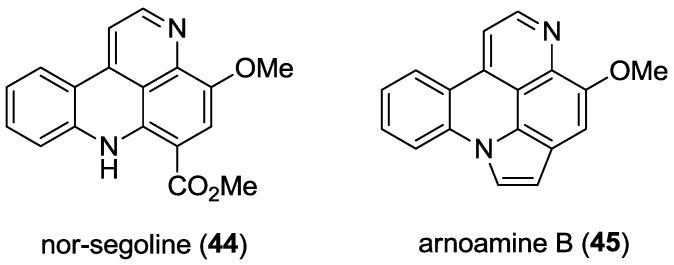
Structures of nor-segoline (**44**) and arnoamine B (**45**).

It has been previously reported that heating styelsamine B (**13**) in MeOH/4 N HCl for 48 h yields the alkylamino analogue styelsamine D (**15**) in quantitative yield [7]. In our hands, repeating this reaction yielded not only **15** (60%) but also a new *O*-methyl analogue **43** in 45% yield (Scheme 4).

(+)-HRESI Mass spectrometric analysis of **43** identified a pseudomolecular ion at *m/z* 292.1448 [M + H]^+^ (calcd for C_18_H_18_N_3_O, 292.1444), being 14 mass units higher than styelsamine D **15**. Detailed analysis of NMR data for **43** and comparison with those data observed for styelsamine D established the presence of an *O*-methyl group [δ_H_ 4.06 (3H, s); δ_C_ 56.9] which was placed at C-11 by observation of an HMBC correlation between δ_H_ 4.06 and δ_C_ 138.7. These chemical shifts agree favorably with the corresponding sub-structural unit of nor-segoline (**44**) (*Eudistoma* sp.) [11] and arnoamine B (**45**) (*Cystodytes* sp.) [12] (Figure 2). Repeating the hydrolysis of styelsamine B, dissolved in MeOH/4N HCl (1:1), but heating at 80 °C for the shorter period of 24 h afforded styelsamine D **15** in 75% yield.

With the unexpected synthesis of **43**, the opportunity was taken to prepare a further subset of *N*-acyl analogues. Thus PyBOP-mediated reaction of **43** with the appropriate carboxylic acid in DMF afforded acrylamide **46** [2] (88% yield) and 2-phenylacetamide **47** (48% yield), while 3-phenylpropanamide **48** was prepared from **43** by reaction with dihydrocinnamoyl chloride in THF (43% yield) (Scheme 4). For each of **46**–**48**, the expected pseudomolecular ion was observed in high resolution ESI-mass spectrometry, and NMR data analysis revealed the expected resonances of the 11-*O*-methylstyelsamine scaffold with expected variation in the NH-14 amide sidechain.

### 2.2. Biological Activities

Previous studies have shown that pyridoacridine alkaloids bind to DNA by a mechanism of base-pair intercalation [13,14]. In the present work, we made use of the fluorescence-based ethidium bromide displacement assay [15] to determine the apparent binding constant (*K*_app_) of the pyridoacridine and pyridoacridine alkaloids. The assay can also be used as an indicator for relative binding affinity, hence, ranking individual compounds. Ethidium bromide exhibits elevated fluorescence (at emission: 546 nm; excitation: 595 nm) when intercalated into DNA, but when displaced by a competing DNA binding agent the observed fluorescence decreases [15].

Using acetate buffer (pH 5), the ability of each of the test compounds to displace ethidium bromide from calf thymus (CT) DNA was measured at a range of concentrations and the data interpolated to determine a C_50_ value (concentration required to reduce the fluorescence by 50%) of each compound. The apparent binding constant (*K*_app_) was then calculated using the formula: *K*_app_ = *K*_ethidium__bromide_ × (1.26/C_50_), where *K*_ethidium__bromide_ = 2.1 × 10^6^ M(bp)^−1^ [16], in order to rank each compound according to their binding affinity (Table 1).

The results in Table 1 show that styelsamines B (**13**) and D (**15**) exhibit the highest affinity for CT-DNA within the styelsamine compound library, with *K*_app_ 5.33 × 10^6^ and 3.64 × 10^6^ M^−1^, respectively. Other styelsamine analogues were revealed to have mild to low affinity for CT-DNA, suggesting that the various sidechains hinder DNA binding. Reinforcing this point was the observation that the palmitamide analogue **38** exhibited the lowest DNA affinity of the styelsamine analogues. A similar trend was observed for the small library of *O*-methyl styelsamine analogues, with *O*-methyl styelsamine D (**43**) exhibiting a higher apparent binding constant, at 4.72 × 10^6^ M^−1^, compared to the other *N*-acyl analogues **46**–**48**. In the case of the cystodytin library, natural products cystodytin A (**1**) and J (**10**) exhibited slightly higher apparent binding constants than their un-natural analogues (entries 12–17). The cystodytin analogues were significantly less soluble in aqueous media than their styelsamine counterparts, with the iminoquinones typically requiring the addition of 0.5% DMSO to facilitate dissolution. In the case of cystodytin J (**10**), addition of 0.5% DMSO to the sample yielded a slightly enhanced DNA binding affinity value (entries 13 and 14). The cystodytin palmitamide analogue **42** could not be solubilized, even with 0.5% DMSO, and so no DNA binding data could be determined.

**Table 1:** DNA binding affinities, antitumor activity and clogP values of styelsamine and cystodytin analogues.

Entry	Compound	C_50_^a^	*K*_app_^b^	One dose ^c^	GI_50_^d^	clogP ^e^
1	**13**	0.50 ± 0.02	5.33	+34.3	3.2 (2.0)	2.6 ± 0.4
2	**15**	0.73 ± 0.02	3.64	+49.7	4.0 (2.2)	2.3 ± 0.2
3	**34 ^f^**	6.62 ± 0.024	0.40	+36.6	3.2 (2.4)	3.6 ± 0.5
4	**35 ^f^**	1.47 ± 0.01	1.80	+10.5	0.63 (1.9)	4.1 ± 0.4
5	**36 ^f^**	4.10 ± 0.47	0.64	nt ^g^		
6	**37 ^f^**	1.67 ± 0.19	1.59	−26.9	0.40 (1.7)	4.4 ± 0.4
7	**38 ^f^**	35.1 ± 1.3	0.08	+79.2	inactive ^h^	8.7 ± 1.3
8	**43**	0.56 ± 0.02	4.72	+24.1	1.6 (1.3)	2.7 ± 0.3
9	**46**	5.43 ± 0.15	0.49	+74.6	inactive ^h^	4.0 ± 0.7
10	**47 ^f^**	2.77 ± 0.26	0.95	nt ^g^		
11	**48**	2.45 ± 0.23	1.08	+45.6	3.2 (2.0)	4.7 ± 0.7
12	**1 ^f^**	11.1 ± 0.3	0.24	+21.7	2.0 (1.5)	3.0 ± 0.6
13	**10**	40.7 ± 1.5 ^g^	0.06	+70.9	inactive ^h^	1.9 ± 0.4
14	**10 ^f^**	16.0 ± 0.2	0.17			
15	**39 ^f^**	78.2 ± 4.6	0.03	+4.9	1.3 (1.8)	3.5 ± 0.5
16	**40 ^f^**	24.8 ± 0.6	0.11	nt ^g^		
17	**41 ^f^**	41.7 ± 2.6	0.06	−14.8	0.32 (2.0)	3.8 ± 0.6
	propamidine	29.1 ^i^	0.09	-	-	-

^a^ C_50_ is defined as the drug concentration (µM), which gives a 50% decrease in the fluorescence of bound ethidium bromide for an [ethidium bromide]:[DNA] molar ratio of 12.6:10. Average and standard error of 3 independent determinations are reported. ^b^ Apparent binding constant (×10^6^ M^−1^); *K*_app_ were calculated as follows: *K*_app_ = (1.26/C_50_) × *K*_ethidium__bromide_, where *K*_ethidium__bromide_ = 2.1 × 10^6^ M^−1^. ^c^ NCI one dose (10 μM) mean growth (%). ^d^ GI_50_ (50% growth inhibition) data (μM) are averaged calculated mean values obtained from two experiments at the NCI. Value in parenthesis is the observed range of data, being the number of log 10 units between the most and least sensitive cell line(s) in the panel. ^e^ cLogP calculated using ALOGPS 2.1, as described in [17,18]. ^f^ Solution prepared in 0.5% DMSO/acetate buffer. ^g^ Not tested. ^h^ Inactive: preliminary one dose evaluation at the NCI indicated the compound was inactive. ^i^ Literature value 23 μM reported in [16].

The library of analogues was submitted to the NCI for evaluation against their panel of human tumor cell lines. Preliminary one dose (10 μM) testing against 57 human tumor cell lines is summarized as a single value, the mean growth inhibition percentage over all cell lines, shown in Table 1. Of the styelsamine analogues **13**, **15**, **34**, **35**, **37**, **38** (entries 1–7), 3-phenylpropanamide **37** was observed to be the most active, with the 10 μM dose resulting in mean cell kill (negative growth). The remaining analogues were considered either mildly active, or inactive in the case of palmitamide **38**. The corresponding *O*-methyl styelsamine analogues **43**, **46** and **48** exhibited moderate to poor growth inhibition (entries 8–11). Of the four cystodytin analogues tested (**1**, **10**, **39**, **41**, entries 12–17), cystodytin J (**10**) was considered inactive, while the 3-phenylpropanamide analogue **41** was observed to be the most active. The wealth of data obtained from even this single dose testing afforded the ability to determine whether these alkaloids exhibit cell line specific activities. Sub-panel selectivity was observed for styelsamine B (**13**, more selective towards melanoma, non-small cell lung cancer, ovarian panels), styelsamine D (**15**, non-small cell lung cancer, CNS, renal), **34** (leukemia, renal), **35** (melanoma, renal), **37** (colon, melanoma, renal) and **38** (colon, renal) (see Supplementary Information). Although generally less potent, similar sub-panel selectivities were observed for the *O*-methyl styelsamine analogues [(**43**, non-small cell lung, renal); (**46**, non-small cell lung); (**48**, non-small lung cell, renal)]. In contrast, pyridoacridones **1**, **10** and **39** were essentially non-selective, while 3-phenylpropanoid **41** exhibited selectivity towards colon, melanoma and renal cancer cell line sub-panels. The analogues that were considered active were then progressed to full 5-dose testing against the complete panel of cell lines, leading to determination of levels of activity corresponding to 50% growth inhibition (GI_50_), total growth inhibition (TGI, cytostatic), or 50% lethality (LC_50_). In general most compounds exhibited poor cytotoxicity, failing to reach LC_50_ or TGI levels of activity, and so only panel average GI_50_ values are reported in Table 1. The GI_50_ values observed were in agreement with the relative activities observed in the one dose testing data, and similar sub-panel selectivities were also observed (data not shown). Thus styelsamine analogues **35** and **37** (GI_50_ 0.63 μM and 0.4 μM respectively) and cystodytin analogue **41** (GI_50_ 0.32 μM) were identified as the most potent tumor cell line growth inhibitors in this study. 

**Figure 3:**
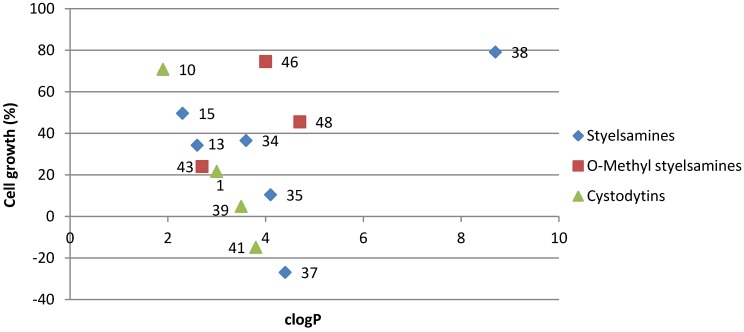
Plot of clogP vs mean cell growth (%) of styelsamine, *O*-methyl styelsamine and cystodytin analogues.

While other groups have reported a direct relationship between cytotoxicity and DNA affinity of pyridoacridine alkaloids [13], the data presented in Table 1 suggests no such correlation between *K_app_* and GI_50_ value for the compounds in the present study. Styelsamines B (**13**), D (**15**) and analogue **34** all exhibited almost the same level of tumor cell growth inhibition (GI_50_ 3.2–4.0 μM), whereas **13** and **15** bound approximately ten times more strongly to DNA than **34**. Also of note is two alkaloids that exhibited approximately the same level of DNA affinity (**38**, *K*_app_0.08 × 10^6^ M^−1^; **41**, 0.06 × 10^6^ M^−1^) exhibited markedly different levels of antiproliferative activity (inactive and GI_50_ 0.32 μM respectively). Cell penetration is clearly a requisite condition for molecules that exert biological activity by targeting DNA, with compound lipophilicity, log P, being a widely accepted descriptor of the ability of a drug to passively diffuse across a membrane. Log P was calculated for each of the test compounds, with the calculations being made using the ALOGPS 2.1 software package [17,18]. The software provides a range of calculated log P values and the average with error is presented in Table 1. Plotting one dose mean cell growth inhibition activities against clogP (Figure 3) identified a correlation for both styelsamine and cystodytin alkaloids, with the best examples of growth inhibition occurring with alkaloid clogP ~4.0–4.5. Interestingly, no such correlation was observed for the, albeit small, data set of *O*-methyl styelsamine analogues **43**, **46** and **48**.

In summary, the current results have identified that natural and un-natural styelsamine and cystodytin analogues exhibit DNA affinity with the aminophenol styelsamines being the more potent series. Lipophilicity was found to be an important determinant of cell-based antiproliferative activity, with optimal activity being observed for alkaloids with clogP 4.0–4.5. The observation of enhanced antiproliferative activity associated with 3-phenylpropanamide analogues **37** and **41** suggests that sidechain modified analogues of styelsamines and/or cystodytins may have potential as new classes of antitumor agents.

## 3. Experimental Section

### 3.1. General

HRMS data were acquired on a Bruker micrOTOF-QII mass spectrometer. Infrared spectra were recorded on a Perkin-Elmer Spectrum 100 Fourier-transform IR spectrometer equipped with a universal ATR accessory. Ultraviolet-visible spectra were run as acetate buffer (pH 5) solutions either on a UV-2101 PC Shimadzu UV-VIS scanning spectrophotometer or a Perkin-Elmer Lambda 35 UV/VIS spectrometer. Fluorescence intensity was recorded on the Perkin-Elmer LS 55 Luminescence spectrometer. NMR spectra were recorded using a Bruker Avance DRX 400 spectrometer operating at 400 MHz for ^1^H nuclei and 100 MHz for ^13^C nuclei. Proto-deutero solvent signals were used as internal references (DMSO-*d*_6_: δ_H_ 2.50, δ_C_ 39.52; CDCl_3_: δ_H_ 7.25, δ_C_ 77.0; CD_3_OD: δ_H_ 3.30, δ_C_ 49.05). Analytical reversed-phase HPLC was run on a Dionex UltiMate 3000RS, using an Alltech platinum C_18_ 3 µm column (33 × 7 mm) and eluting with a linear gradient of H_2_O (0.05% TFA) to MeCN over 13.5 min at 2 mL/min. Flash column chromatography was performed using reversed-phase Merck Lichroprep RP-2 or RP-18, Kieselgel 60 PF silica gel, or by size exclusion chromatography on Pharmacia Biotech Sephadex LH-20. Thin layer chromatography used 0.2 mm thick plates of Kiesegel F_254_ (Merck, Manakau, New Zealand).

### 3.2. Synthetic Procedures

#### 3.2.1. Tryptamine Methyl Carbamate (**17**)

To a stirred solution of tryptamine (2.0 g, 0.01 mol) in a degassed biphasic mixture of NaOH (1 N, 12.5 mL) and EtOAc (20 mL) was added methyl chloroformate (1.01 mL, 13.13 mmol) dropwise, under N_2_. The brown solution was stirred for 30 min at room temperature, after which it was washed with H_2_O (2 × 40 mL) and the organic phase dried *in vacuo*. The residue was dissolved in EtOAc (10 mL) and then added to hexane (50 mL) to yield **17** as brown crystals (2.47 g, 73% yield).

Mp 82.9–83.9 °C (lit. [19] 79.0–81.0 °C); *R*_f_ (1 Hex:2 EtOAc) 0.64; IR ν_max_ (ATR) 3400, 1686, 1544, 1264 cm^−1^; ^1^H NMR (CDCl_3_, 400 MHz) δ_H_ 8.02 (1H, br s, NH-1), 7.61 (1H, d, *J* = 7.4 Hz, H-4), 7.38 (1H, d, *J* = 7.6 Hz, H-7), 7.21 (1H, td, *J* = 7.6, 1.2 Hz, H-6), 7.14 (1H, td, *J* = 7.4, 0.9 Hz, H-5), 7.03 (1H, d, *J* = 2.1 Hz, H-2), 4.73 (1H, br s, NH-10), 3.65 (3H, s, OMe), 3.53 (2H, dt, *J* = 6.8, 6.8 Hz, H_2_-9), 2.98 (2H, t, *J* = 6.8 Hz, H_2_-8); ^13^C NMR (CDCl_3_, 100 MHz) δ_C_ 157.1 (C-11), 136.4 (C-7a), 127.2 (C-3a), 122.2 (C-6 or C-2), 122.0 (C-6 or C-2), 119.4 (C-5), 118.7 (C-4), 112.9 (C-3), 111.2 (C-7), 52.0 (OMe), 41.2 (C-9), 25.8 (C-8); (+)-ESIMS *m/z* 219 [M + H]^+^; (+)-HRESIMS [M + H]^+^ 219.1131 (calcd. for C_12_H_15_N_2_O_2_, 219.1128). ^1^H and ^13^C NMR data agreed with literature [19].

#### 3.2.2. Kynuramine Methyl Carbamate (**18**) and *N*-Acetyl-kynuramine Methyl Carbamate (**19**)

Ozone was bubbled into a solution of tryptamine methyl carbamate (**17**) (1.00 g, 4.59 mmol) in AcOH (20 mL) that was stirred in an ice bath. The reaction was stopped once the solution became frozen. The frozen solution was degassed with N_2_ for 5 min and then conc. HCl (1 mL) was added to the solution and warmed to 40 °C for 1.5 h. After this time the solution was dried *in vacuo*, the residue dissolved in CH_2_Cl_2_ (20 mL), and washed with phosphate buffer until neutral (3 × 20 mL). The organic phase was dried (MgSO_4_), solvent removed *in vacuo* and the mixture purified using silica gel flash chromatography (hexane/EtOAc) to afford kynuramine methyl carbamate **18** as a yellow solid (0.42 g, 42% yield) and **19** also as a yellow solid (0.13 g, 10% yield).

*Kynuramine methyl carbamate* **18**: Mp 90.0–91.0 °C (lit. [20] 98.0–99.0 °C); *R*_f_ (10% MeOH/CH_2_Cl_2_) 0.89; IR ν_max_ (ATR) 3360, 1685, 1619, 1531, 1264 cm^−1^; ^1^H NMR (CDCl_3_, 400 MHz) δ_H_ 7.68 (1H, d, *J* = 7.6 Hz, H-6), 7.26 (1H, dt, *J* = 7.6, 1.6 Hz, H-4), 6.65–6.61 (2H, m, H-3 and H-5), 3.64 (3H, s, OMe), 3.57 (2H, dt, *J* = 5.6, 5.6 Hz, H_2_-10), 3.17 (2H, t, *J* = 5.6 Hz, H_2_-9); ^13^C NMR (CDCl_3_, 100 MHz) δ_C_ 201.1 (C-8), 157.1 (C-12), 150.4 (C-2), 134.6 (C-4), 131.0 (C-6), 117.7 (C-5), 117.4 (C-7), 115.9 (C-3), 52.0 (OMe), 38.9 (C-9), 36.2 (C-10); (+)-ESIMS *m/z* 223 [M + H]^+^; (+)-HRESIMS [M + H]^+^ 223.1076 (calcd. for C_11_H_15_N_2_O_3_, 223.1077).

*N-Acetyl kynuramine methyl carbamate* **19**: Mp 120.0–121.0 °C; *R*_f_ (1 Hex:2 EtOAc) 0.30; IR ν_max_ (ATR) 3332, 3220, 3112, 2947, 1700, 1686, 1544, 1292, 1195, 760 cm^−1^; ^1^H NMR (CDCl_3_, 400 MHz) δ_H_ 11.62 (1H, br s, NH-1), 8.70 (1H, d, *J* = 7.3 Hz, H-3), 7.90 (1H, d, *J* = 6.8 Hz, H-6), 7.55 (1H, td, *J* = 7.3, 1.5 Hz, H-4), 7.11 (1H, td, *J* = 6.8, 1.3 Hz, H-5), 5.29 (1H, br s, NH-11), 3.66 (3H, s, OMe), 3.58 (2H, dt, *J* = 5.6, 5.6 Hz, H_2_-10), 3.29 (2H, t, *J* = 5.6 Hz, H_2_-9), 2.23 (3H, s, H_3_-14); ^13^C NMR (CDCl_3_, 100 MHz) δ_C_ 203.4 (C-8), 169.4 (C-13), 157.0 (C-12), 141.1 (C-2), 135.4 (C-4), 130.8 (C-6), 122.4 (C-5), 121.1 (C-7), 120.8 (C-3), 52.1 (OMe), 39.8 (C-9), 36.0 (C-10), 25.6 (C-14); (+)-ESIMS *m/z* 265 [M + H]^+^; (+)-HRESIMS [M + H]^+^ 265.1191 (calcd. for C_13_H_17_N_2_O_4_, 265.1183).

An alternative method to bypass the formation of acetamide **19** was to take the crude reaction product containing both **18** and **19**, dissolve it in aq. HCl (10%, 40 mL), and heat at reflux for 4 h. Removal of solvents *in vacuo* afforded **18** as a yellow solid (1.35 g, 66% yield over two steps).

#### 3.2.3. Kynuramine Dihydrobromide (**20**)

A solution of kynuramine methyl carbamate **18** (1.346 g, 6.06 mmol) in HBr saturated AcOH (20 mL) was heated to 80 °C and stirred for 18 h under N_2_. The brown solution was cooled to room temperature and THF (80 mL) was added which resulted in the formation of a precipitate. The mixture was stirred in an ice bath for 20 min, then filtered. The brown solid was dried under N_2_ to afford **20** (1.89 g, 96% yield).

Mp 192.0–193.0 °C (lit. [21] 214.0–216.0 °C); IR ν_max_ (ATR) 3400, 1705, 1619, 1543, 1261 cm^−1^; ^1^H NMR (CD_3_OD, 400 MHz) δ_H_ 8.14 (1H, dd, *J* = 7.8, 1.4 Hz, H-6), 7.69 (1H, td, *J* = 7.9, 1.4 Hz, H-4), 7.51 (1H, td, *J* = 7.8, 1.1 Hz, H-5), 7.42 (1H, dd, *J* = 7.9, 1.1 Hz, H-3), 3.55 (2H, t, *J* = 6.4 Hz, H_2_-9), 3.34 (2H, t, *J* = 6.4 Hz, H_2_-10); ^13^C NMR (CD_3_OD, 100 MHz) δ_C_ 200.8 (C-8), 162.8 (C-2), 136.3 (C-4), 132.8 (C-6), 128.9 (C-5), 128.5 (C-7), 125.4 (C-3), 37.8 (C-9), 35.9 (C-10); (+)-ESIMS *m/z* 165 [M + H]^+^; (+)-HRESIMS [M + H]^+^ 165.1016 (calcd. for C_9_H_13_N_2_O, 165.1022). 

#### 3.2.4. *N*-(3,4-Dimethoxyphenethyl)acetamide (**22**)

Et_3_N (1.54 mL, 0.01 mol) and acetic anhydride (1.56 mL, 0.02 mol) was added to 2-(3,4-dimethoxyphenyl)ethylamine (**21**) (0.93 mL, 5.52 mmol). The reaction mixture was yellow, and was stirred at room temperature for 1 h under N_2_. CH_2_Cl_2_ (100 mL) was added then washed with H_2_O (50 mL) and the organic phase dried *in vacuo*, to afford **22** as a yellow solid (1.17 g, 95% yield).

Mp 97.8–98.6 °C (lit. [22] 100.0–101.0 °C); *R*_f_ (5% MeOH/CH_2_Cl_2_) 0.39; IR ν_max_ (ATR) 3250, 3080, 2928, 2840, 1631, 1590, 1516, 1261, 1232, 1155, 1019 cm^−1^; ^1^H NMR (CDCl_3_, 400 MHz) δ_H_ 6.80 (1H, d, *J* = 8.0 Hz, H-8), 6.72 (1H, d, *J* = 1.8 Hz, H-5), 6.70 (1H, dd, *J* = 8.0, 1.8 Hz, H-9), 5.55 (1H, br s, NH-1), 3.87 (6H, s, OMe), 3.30 (2H, dt, *J* = 7.0, 7.0 Hz, H_2_-2), 2.60 (2H, t, *J* = 7.0 Hz, H_2_-3), 1.92 (3H, s, H_3_-11); ^13^C NMR (CDCl_3_, 100 MHz) δ_C_ 170.0 (C-10), 149.1 (C-6), 147.7 (C-7), 131.3 (C-4), 120.6 (C-9), 111.9 (C-8), 111.4 (C-5), 55.9 (OMe × 2), 40.7 (C-2), 35.2 (C-3), 23.3 (C-11); (+)-ESIMS *m/z* 224 [M + H]^+^; (+)-HRESIMS [M + H]^+^ 224.1279 (calcd. for C_12_H_18_NO_3_, 224.1281). ^1^H and ^13^C NMR data agreed with literature [22]. 

#### 3.2.5. *N*-(3,4-Dimethoxyphenethyl)-3-methylbut-2-enamide (**23**)

To a solution of 3,3-dimethylacrylic acid (100 mg, 1.00 mmol) in dry DMF (3 mL) was added 2-(3,4-dimethoxyphenyl)ethylamine (**21**) (0.17 mL, 1.00 mmol), PyBOP (520 mg, 1.00 mmol) and Et_3_N (0.42 mL, 3.00 mmol). The mixture was stirred under N_2_ at room temperature for 20 h. CH_2_Cl_2_ (20 mL) was added, washed with H_2_O (30 mL), and the organic phase dried *in vacuo*. EtOAc (75 mL) was added and washed with 5% aq. K_2_CO_3_ (50 mL), 10% HCl (10 mL) and brine (20 mL). The organic phase was then dried (MgSO_4_) and solvent removed *in vacuo* to give **23** as an orange-brown solid (0.24 g, 92% yield).

Mp 67.0–68.2 °C; *R*_f_ (5% MeOH/CH_2_Cl_2_) 0.22; IR ν_max_ (ATR) 3302, 3002, 2939, 1666, 1141, 1027 cm^−1^; ^1^H NMR (CDCl_3_, 400 MHz) δ_H_ 6.79 (1H, d, *J* = 8.4 Hz, H-8), 6.73–6.71 (2H, m, H-5 and H-9), 5.48 (1H, s, H-11), 3.84 (6H, s, OMe), 3.50 (2H, dt, *J* = 7.2, 7.2 Hz, H_2_-2), 2.76 (2H, t, *J* = 7.2 Hz, H_2_-3), 2.12 (3H, s, H_3_-14), 1.79 (3H, s, H_3_-13); ^13^C NMR (CDCl_3_, 100 MHz) δ_C_ 166.9 (C-10), 150.8 (C-12), 148.9 (C-6), 147.5 (C-7), 131.5 (C-4), 120.6 (C-9), 118.4 (C-11), 111.9 (C-5), 111.3 (C-8), 55.8 (OMe × 2), 40.3 (C-2), 35.3 (C-3), 27.0 (C-13), 19.7 (C-14); (+)-ESIMS *m/z* 264 [M + H]^+^; (+)-HRESIMS [M + H]^+^ 264.1595 (calcd. for C_15_H_22_NO_3_, 264.1594).

#### 3.2.6. *N*-(3,4-Dimethoxyphenethyl)benzamide (**24**)

To a cold (0 °C) solution of 2-(3,4-dimethoxyphenyl)ethylamine (**21**) (0.093 mL, 0.55 mmol) and Et_3_N (0.35 mL, 2.48 mmol) in THF (7.0 mL) was added benzoyl chloride (0.223 mL, 1.93 mmol). The milky white solution was warmed to room temperature and solvents were removed *in vacuo*. CHCl_3_ (20 mL) was added, the solution washed with 10% aq. NaCO_3_ (50 mL), H_2_O (20 mL) and brine (20 mL) and then the organic phase was dried *in vacuo*. The residue was triturated with hexane (6 mL) and EtOAc (2 mL) to give **24** as a greenish-white solid (90 mg, 57% yield). 

Mp 85.0–85.8 °C (lit. [23] 85.0–86.0 °C); *R*_f_ (5% MeOH/CH_2_Cl_2_) 0.67; IR ν_max_ (ATR) 3236, 2981, 1634, 1590, 1231 cm^−1^; ^1^H NMR (CDCl_3_, 400 MHz) δ_H_ 7.71–7.69 (2H, m, H-12 and H-16), 7.44 (1H, tt, *J* = 6.4, 1.2 Hz, H-14), 7.38–7.34 (2H, m, H-13 and H-15), 6.78 (1H, d, *J* = 8.0 Hz, H-8), 6.74–6.72 (2H, m, H-5 and H-9), 3.82 (3H, s, OMe), 3.79 (3H, s, OMe), 3.66 (2H, t, *J* = 7.2 Hz, H_2_-2), 2.85 (2H, t, *J* = 7.2 Hz, H_2_-3); ^13^C NMR (CDCl_3_, 100 MHz) δ_C_ 167.6 (C-10), 148.9 (C-6), 147.5 (C-7), 134.2 (C-11), 131.4 (C-14), 131.3 (C-4), 128.4 (C-13 and C-15), 126.8 (C-12 and C-16), 120.6 (C-9), 111.9 (C-5), 111.3 (C-8), 55.7 (OMe × 2), 41.3 (C-2), 35.1 (C-3); (+)-ESIMS *m/z* 286 [M + H]^+^; (+)-HRESIMS [M + H]^+^ 286.1437 (calcd. for C_17_H_20_NO_3_, 286.1438). ^1^H NMR data agreed with literature [23].

#### 3.2.7. *N*-(3,4-Dimethoxyphenethyl)-2-phenylacetamide (**25**)

To a solution of phenylacetic acid (200 mg, 1.47 mmol) in dry DMF (3 mL) was added 2-(3,4-dimethoxyphenyl)ethylamine (**21**) (0.25 mL, 1.47 mmol), PyBOP (764 mg, 1.47 mmol) and Et_3_N (0.62 mL, 4.41 mmol). The mixture was stirred under N_2_ at room temperature for 26 h. CH_2_Cl_2_ (20 mL) was added, washed with H_2_O (30 mL), and the organic phase dried *in vacuo*. The crude product was triturated with hexane (15 mL) and EtOAc (7 mL) to yield a white solid that was recrystallized from ethanol (3 mL) to afford **25** as white crystals (0.438 g, 99% yield).

Mp 111.1–112.2 °C (lit. [24] 110–111 °C); *R*_f_ (3 EtOAc:1 Hex) 0.40; IR ν_max_ (ATR) 3245, 3008, 2994, 1660, 1605, 1232 cm^−1^; ^1^H NMR (CDCl_3_, 400 MHz) δ_H_ 7.32–7.25 (3H, m, H-14, H-15 and H-16), 7.16 (2H, dd, *J* = 8.0, 2.0 Hz, H-13 and H-17), 6.71 (1H, d, *J* = 8.2 Hz, H-8), 6.59 (1H, d, *J* = 2.0 Hz, H-5), 6.54 (1H, dd, *J* = 8.2, 2.0 Hz, H-9), 5.36 (1H, br s, NH-1), 3.85 (3H, s, OMe), 3.81 (3H, s, OMe), 3.53 (2H, s, H_2_-11), 3.44 (2H, dt, *J* = 6.9, 6.9 Hz, H_2_-2), 2.67 (2H, t, *J* = 6.9 Hz, H_2_-3); ^13^C NMR (CDCl_3_, 100 MHz) δ_C_ 170.9 (C-10), 149.0 (C-6 or C-7), 147.6 (C-6 or C-7), 134.8 (C-12), 131.1 (C-4), 129.4 (C-13 and C-17), 129.0 (C-14 and C-16), 127.3 (C-15), 120.5 (C-9), 111.7 (C-5), 111.3 (C-8), 55.9 (OMe), 55.8 (OMe), 43.9 (C-11), 40.7 (C-2), 35.0 (C-3); (+)-ESIMS *m/z* 300 [M + H]^+^; (+)-HRESIMS [M + H]^+^ 300.1593 (calcd. for C_18_H_22_NO_3_, 300.1594). ^1^H NMR data agreed with literature [24].

#### 3.2.8. *N*-(3,4-Dimethoxyphenethyl)-3-phenylpropanamide (**26**)

To a cold (0 °C) solution of 2-(3,4-dimethoxyphenyl)ethylamine (**21**) (90 μL, 0.55 mmol) and Et_3_N (0.35 mL, 2.48 mmol) in THF (4.5 mL) was added dihydrocinnamoyl chloride (0.29 mL, 1.93 mmol). The milky white solution was warmed to room temperature and then the solvents were removed *in vacuo*. CHCl_3_ (20 mL) was added, the solution washed with 10% aq. NaCO_3_ (50 mL), H_2_O (20 mL) and brine (20 mL) and then the organic phase was dried *in vacuo*. The residue was triturated with hexane (7 mL) and EtOAc (2 mL) to give **26** as a pale yellow solid (0.16 g, 93% yield).

Mp 123.1–124.0 °C; *R*_f_ (5% MeOH/CH_2_Cl_2_) 0.45; IR ν_max_ (ATR) 3249, 2974, 1631, 1534, 1232 cm^−1^; ^1^H NMR (CDCl_3_, 400 MHz) δ_H_ 7.29–7.26 (2H, m, H-15, H-17), 7.24–7.16 (3H, m, H-14, H-16, H-18), 6.77 (1H, d, *J* = 8.0 Hz, H-8), 6.65 (1H, d, *J* = 2.0 Hz, H-5), 6.61 (1H, dd, *J* = 8.0, 2.0 Hz, H-9), 3.85 (3H, s, OMe), 3.84 (3H, s, OMe), 3.45 (2H, dt, *J* = 7.2, 7.2 Hz, H_2_-2), 2.94 (2H, t, *J* = 8.0 Hz, H_2_-12), 2.68 (2H, t, *J* = 7.2 Hz, H_2_-3), 2.42 (2H, t, *J* = 8.0 Hz, H_2_-11); ^13^C NMR (CDCl_3_, 100 MHz) δ_C_ 172.0 (C-10), 149.0 (C-6), 147.7 (C-7), 141.0 (C-13), 131.3 (C-4), 128.6 (C-15 and C-17), 128.3 (C-14 and C-18), 126.2 (C-16), 120.6 (C-9), 111.8 (C-5), 111.3 (C-8), 55.9 (OMe × 2), 40.6 (C-2), 38.5 (C-11), 35.2 (C-3), 31.7 (C-12); (+)-ESIMS *m/z* 314 [M + H]^+^; (+)-HRESIMS [M + H]^+^ 314.1748 (calcd. for C_19_H_24_NO_3_, 314.1751).

#### 3.2.9. *N*-(3,4-Dimethoxyphenethyl)palmitamide (**27**)

To a cold (0 °C) solution of 2-(3,4-dimethoxyphenyl)ethylamine (**21**) (0.20 mL, 1.10 mmol) and Et_3_N (0.70 mL, 5.0 mmol) in THF (10 mL) was added palmitoyl chloride (1.18 mL, 3.86 mmol). The milky white solution was warmed to room temperature and then the solvents were removed *in vacuo*. CHCl_3_ (20 mL) was added, the solution washed with 10% aq. NaCO_3_ (50 mL), H_2_O (20 mL) and brine (20 mL) and then the organic phase was dried *in vacuo*. The residue was triturated with hexane (10 mL) and EtOAc (5 mL) to give **27** as a white solid (0.30 g, 65% yield).

Mp 94.0–95.1 °C; *R*_f_ (3 EtOAc:1 Hex) 0.73; IR ν_max_ (ATR) 3301, 2955, 2918, 1705, 1638, 1591, 1232, 1140 cm^−1^; ^1^H NMR (CDCl_3_, 400 MHz) δ_H_ 6.80 (1H, d, *J* = 8.4 Hz, H-8), 6.73–6.71 (2H, m, H-5 and H-9), 5.43 (1H, s, NH-1), 3.86 (6H, s, OMe), 3.49 (2H, dt, *J* = 7.2, 7.2 Hz, H_2_-2), 2.75 (2H, t, *J* = 7.2 Hz, H_2_-3), 2.11 (2H, t, *J* = 7.6 Hz, H_2_-11), 1.58 (2H, br t, *J* = 7.2 Hz, H_2_-12), 1.24 (24H, br s, H_2_-13–H_2_-24), 0.87 (3H, t, *J* = 7.2 Hz, H_3_-25); ^13^C NMR (CDCl_3_, 100 MHz) δ_C_ 173.2 (C-10), 149.2 (C-6), 147.8 (C-7), 131.6 (C-4), 120.8 (C-9), 112.0 (C-5), 111.4 (C-8), 56.0 (OMe × 2), 40.7 (C-2), 37.0 (C-11), 35.5 (C-3), 32.1 (C-23), 29.8 (C-13–C-22), 25.9 (C-12), 22.8 (C-24), 14.3 (C-25); (+)-ESIMS *m/z* 420 [M + H]^+^; (+)-HRESIMS [M + H]^+^ 420.3460 (calcd. for C_26_H_45_NO_3_, 420.3472).

#### 3.2.10. General Procedure for the Preparation of *N*-Acyl Dopamine Analogues **28–33**

To a stirred solution of 3,4-dimethoxyphenethylamide **28**–**33** in dry CH_2_Cl_2_ (20 mL) in a salted ice bath, boron tribromide (10 equiv.) in dry CH_2_Cl_2_ (10 mL) was added dropwise. The solution turned from yellow to orange, and was stirred under N_2_ for 20 h with temperature rising to room temperature. MeOH (3 mL) and saturated brine (5 mL) were then added dropwise. EtOAc (30 mL) was added, the organic phase washed with H_2_O (30 mL), dried (MgSO_4_) and solvent removed *in vacuo* to afford the *N*-acyl dopamine analogue. The product was used in the subsequent reaction without further purification.

##### 3.2.10.1. *N*-Acetyl Dopamine (**28**)

From *N*-(3,4-dimethoxyphenethyl)acetamide (**22**) (200 mg, 0.9 mmol) to afford **28** as a yellow oil (158 mg, 90% yield).

IR ν_max_ (ATR) 3215, 1624, 1558, 1439, 1284 cm^−1^; ^1^H NMR (CD_3_OD, 400 MHz) δ_H_ 6.69 (1H, d, *J* = 8.0 Hz, H-8), 6.65 (1H, d, *J* = 2.0 Hz, H-5), 6.45 (1H, dd, *J* = 8.0, 2.0 Hz, H-9), 3.30 (2H, t, *J* = 7.2 Hz, H_2_-2), 2.60 (2H, t, *J* = 7.2 Hz, H_2_-3), 1.89 (3H, s, H_3_-11); ^13^C NMR (CD_3_OD, 100 MHz) δ_C_ 172.1 (C-10), 144.8 (C-6), 143.3 (C-7), 130.7 (C-4), 119.8 (C-9), 115.6 (C-8), 115.1 (C-5), 41.0 (C-2), 34.4 (C-3), 21.2 (C-11); (+)-ESIMS *m/z* 196 [M + H]^+^; (+)-HRESIMS [M + H]^+^ 196.0970 (calcd. for C_10_H_14_NO_3_, 196.0968).

##### 3.2.10.2. *N*-(3,4-Dihydroxyphenethyl)-3-methylbut-2-enamide (**29**)

From *N*-(3,4-dimethoxyphenethyl)-3-methylbut-2-enamide (**23**) (90 mg, 0.35 mmol) to afford **29** as a yellow oil (81 mg, 98% yield).

IR ν_max_ (ATR) 3240, 2976, 1657, 1598, 1442, 1165 cm^−1^; ^1^H NMR (CD_3_OD, 400 MHz) δ_H_ 6.77 (1H, d, *J* = 8.0 Hz, H-8), 6.65 (1H, d, *J* = 2.0 Hz, H-5), 6.51 (1H, dd, *J* = 8.0, 2.0 Hz, H-9), 5.50 (1H, s, H-11), 3.30 (2H, t, *J* = 8.0 Hz, H_2_-2), 2.62 (2H, t, *J* = 8.0 Hz, H_2_-3), 2.06 (3H, s, H_3_-14), 1.80 (3H, s, H_3_-13); ^13^C NMR (CD_3_OD, 100 MHz) δ_C_ 169.7 (C-10), 151.4 (C-12), 146.2 (C-6), 144.7 (C-7), 132.2 (C-4), 121.0 (C-9), 119.6 (C-11), 116.9 (C-5), 116.3 (C-8), 42.1 (C-2), 36.1 (C-3), 27.2 (C-13), 20.0 (C-14); (+)-ESIMS *m/z* 236 [M + H]^+^; (+)-HRESIMS [M + H]^+^ 236.1275 (calcd. for C_13_H_18_NO_3_, 236.1281).

##### 3.2.10.3. *N*-(3,4-Dihydroxyphenethyl)benzamide (**30**)

From *N*-(3,4-dimethoxyphenethyl)benzamide (**24**) (196 mg, 0.69 mmol) to afford **30** as a yellow oil (150 mg, 85% yield).

IR ν_max_ (ATR) 3251, 2257, 1634, 1529, 1285 cm^−1^; ^1^H NMR (DMSO-*d*_6_, 400 MHz) δ_H_ 8.49 (1H, t, *J* = 5.6 Hz, NH-1), 7.83–7.80 (2H, m, H-12 and H-16), 7.51 (1H, tt, *J* = 7.2, 1.6 Hz, H-14), 7.45 (2H, td, *J* = 6.8, 1.6 Hz, H-13 and H-15), 6.65–6.62 (2H, m, H-5 and H-8), 6.47 (1H, dd, *J* = 8.0, 2.0 Hz, H-9), 3.39 (2H, dt, *J* = 8.0, 5.6 Hz, H_2_-2), 2.65 (2H, t, *J* = 8.0 Hz, H_2_-3); ^13^C NMR (DMSO-*d*_6_, 100 MHz) δ_C_ 166.0 (C-10), 145.0 (C-6), 143.4 (C-7), 134.6 (C-11), 130.9 (C-14), 130.2 (C-4), 128.2 (C-12 and C-16), 127.0 (C-13 and C-15), 119.2 (C-9), 115.9 (C-5), 115.4 (C-8), 41.2 (C-2), 34.6 (C-3); (+)-ESIMS *m/z* 258 [M + H]^+^; (+)-HRESIMS [M + H]^+^ 258.112 (calcd. for C_15_H_16_NO_3_, 258.1125).

##### 3.2.10.4. *N*-(3,4-Dihydroxyphenethyl)-2-phenylacetamide (**31**)

From *N*-(3,4-dimethoxyphenethyl)-2-phenylacetamide (**25**) (200 mg, 0.67 mmol) to afford **31** as a yellow oil (142 mg, 79% yield). 

IR ν_max_ (ATR) 3546, 3399, 3236, 1636, 1613, 1524, 1495, 1358, 1282, 1193 cm^−1^; ^1^H NMR (CD_3_OD, 400 MHz) δ_H_ 7.31–7.26 (2H, m, H-14, H-16), 7.24–7.30 (3H, m, H-13, H-15 and H-17), 6.65 (1H, d, *J* = 8.0 Hz, H-8), 6.62 (1H, d, *J* = 2.0 Hz, H-5), 6.45 (1H, dd, *J* = 8.0, 2.0 Hz, H-9), 3.46 (2H, s, H_2_-11), 3.36–3.33 (2H, m, H_2_-2), 2.62 (2H, t, *J* = 7.2 Hz, H_2_-3); ^13^C NMR (CD_3_OD, 100 MHz) δ_C_ 174.1 (C-10), 146.2 (C-6), 144.8 (C-7), 136.5 (C-12), 131.9 (C-4), 130.1 (C-13 and C-17), 129.6 (C-14 and C-16), 127.9 (C-15), 121.2 (C-9), 116.7 (C-5), 116.4 (C-8), 43.9 (C-11), 42.4 (C-2), 35.8 (C-3); (+)-ESIMS *m/z* 272 [M + H]^+^; (+)-HRESIMS [M + H]^+^ 272.1272 (calcd. for C_16_H_18_NO_3_, 272.1281). ^1^H NMR data agreed with literature [25]. 

##### 3.2.10.5. *N*-(3,4-Dihydroxyphenethyl)-3-phenylpropanamide (**32**)

From *N*-(3,4-dimethoxyphenethyl)-3-phenylpropanamide (**26**) (298 mg, 0.95 mmol) to afford **32** as a yellow oil (240 mg, 89% yield).

IR ν_max_ (ATR) 3214, 1604, 1521, 1446, 1281, 1190 cm^−1^; ^1^H NMR (CD_3_OD, 400 MHz) δ_H_ 7.15 (2H, dt, *J* = 8.4, 0.8 Hz, H-15, H-17), 7.08–7.06 (3H, m, H-14, H-16 and H-18), 6.60 (1H, d, *J* = 8.0 Hz, H-8), 6.56 (1H, d, *J* = 2.4 Hz, H-5), 6.37 (1H, dd, *J* = 8.0, 2.4 Hz, H-9), 3.22–3.20 (2H, m, H_2_-2), 2.78 (2H, t, *J* = 8.2 Hz, H_2_-12), 2.48 (2H, t, *J* = 7.6 Hz, H_2_-3), 2.39 (2H, t, *J* = 8.2 Hz, H_2_-11); ^13^C NMR (CDCl_3_, 100 MHz) δ_C_ 175.6 (C-10), 146.1 (C-6), 144.6 (C-7), 141.8 (C-13), 131.9 (C-4), 129.5 (C-14, C-15, C-17, and C-18), 127.3 (C-16), 121.1 (C-9), 116.8 (C-5), 116.4 (C-8), 42.5 (C-2), 38.7 (C-11), 35.7 (C-3), 33.0 (C-12); (+)-ESIMS *m/z* 286 [M+H]^+^; (+)-HRESIMS [M+H]^+^ 286.1433 (calcd. for C_17_H_20_NO_3_, 286.1438). ^1^H NMR data agreed with literature [25]. 

##### 3.2.10.6. *N*-(3,4-Dihydroxyphenethyl)palmitamide (**33**)

From *N*-(3,4-dimethoxyphenethyl)palmitamide (**27**) (200 mg, 0.48 mmol) to afford **33** as a colourless oil (150 mg, 75% yield).

IR ν_max_ (ATR) 3546, 3401, 3237, 2918, 2059, 1637, 1554, 1356, 1194, 1119 cm^−1^; ^1^H NMR (DMSO-*d*_6_, 400 MHz) δ_H_ 7.78 (1H, t, *J* = 5.6 Hz, NH-1), 6.61 (1H, d, *J* = 8.0 Hz, H-8), 6.55 (1H, d, *J* = 2.5 Hz, H-5), 6.41 (1H, dd, *J* = 8.0, 2.5 Hz, H-9), 3.14 (2H, dt, *J* = 7.2, 5.6 Hz, H_2_-2), 2.50 (2H, t, *J* = 7.2 Hz, H_2_-3), 2.01 (2H, t, *J* = 7.6 Hz, H_2_-11), 1.45 (2H, t, *J* = 6.8 Hz, H_2_-12), 1.23 (24H, br s, H_2_-13–H_2_-24), 0.87 (3H, t, *J* = 7.2 Hz, H_3_-25); ^13^C NMR (DMSO-*d*_6_, 100 MHz) δ_C_ 171.8 (C-10), 144.9 (C-6), 143.4 (C-7), 130.2 (C-4), 119.1 (C-9), 115.8 (C-5), 115.3 (C-8), 40.4 (C-2), 35.4 (C-11), 34.7 (C-3), 31.2 (C-23), 28.7 (C-13–C-22), 25.2 (C-12), 22.0 (C-24), 13.9 (C-25); (+)-ESIMS *m/z* 391 [M + H]^+^; (+)-HRESIMS [M + H]^+^ 392.3156 (calcd. for C_24_H_42_NO_3_, 392.3159). ^1^H NMR data agreed with literature [26]. 

#### 3.2.11. General Procedure for the Preparation of *N*-Acyl Styelsamine Analogues **13**, **34–38**

To a solution of *N*-acyl dopamine (1 equiv.) in degassed 2:1 MeOH:AcOH (6 mL) was added kynuramine dihydrobromide (1.05 equiv.) followed by CeCl_3_·7H_2_O (0.2 equiv.). To the stirred yellow solution under N_2_ was added Ag_2_O (2–4 equiv.) and the suspension warmed to 40 °C for 1.5 h. The yellow solution was filtered and added dropwise to stirring HCl (6 N, 15 mL) at 90 °C and heated for a further 30 min during which time the colour of the solution changed to purple. The solution was dried *in vacuo* and the residue purified by either RP-2 or RP-18 column chromatography using H_2_O (0.05% TFA)–MeOH solvent mixtures to afford the product as a purple oil.

##### 3.2.11.1. Styelsamine B Trifluoroacetate (**13**)

Using the general procedure, reaction of *N*-acetyl dopamine (**28**) (52 mg, 0.27 mmol) with kynuramine dihydrobromide (92 mg, 0.28 mmol), CeCl_3_.7H_2_O (14.0 mg, 0.04 mmol) and Ag_2_O (123 mg, 0.53 mmol) afforded, after RP-2 column chromatography, **13** as a purple oil (22.0 mg, 19% yield).

IR ν_max_ (ATR) 3389, 3075, 1679, 1205, 1138 cm^−1^; *R*_t_ = 5.99 min; ^1^H NMR (CD_3_OD, 400 MHz) δ_H_ 7.93 (1H, d, *J* = 5.8 Hz, H-2), 7.81 (1H, d, *J* = 7.8 Hz, H-4), 7.52 (1H, t, *J* = 8.0 Hz, H-6), 7.46 (1H, d, *J* = 8.0 Hz, H-7), 7.20 (1H, s, H-10), 7.14 (1H, d, *J* = 5.8 Hz, H-3), 7.10 (1H, t, *J* = 7.8 Hz, H-5), 3.20 (2H, t, *J* = 6.8 Hz, H_2_-13), 2.79 (2H, t, *J* = 6.8 Hz, H_2_-12), 2.04 (3H, s, H_3_-16); ^13^C NMR (CD_3_OD, 100 MHz) δ_C_ 174.7 (C-15), 150.8 (C-3a), 143.4 (C-2), 142.3 (C-7a), 138.0 (C-11), 136.2 (C-6), 129.7 (C-8a), 127.2 (C-11a), 126.0 (C-4), 123.9 (C-5), 122.6 (C-10), 121.6 (C-11b), 118.9 (C-7), 117.3 (C-9), 115.2 (C-3b), 105.6 (C-3), 39.3 (C-13), 31.9 (C-12), 22.5 (C-16); (+)-ESIMS *m/z* 320 [M + H]^+^; (+)-HRESIMS [M + H]^+^ 320.1391 (calcd. for C_19_H_18_N_3_O_2_, 320.1394). ^1^H and ^13^C NMR data agreed with literature [6]. 

##### 3.2.11.2. Styelsamine-*N*^14^-3-methyl-but-2-enamide (**34**)

Using the general procedure, reaction of *N*-(3,4-dihydroxyphenethyl)-3-methylbut-2-enamide (**29**) (98 mg, 0.42 mmol) with kynuramine dihydrobromide (142 mg, 0.44 mmol), CeCl_3_·7H_2_O (22 mg, 0.06 mmol) and Ag_2_O (360 mg, 1.57 mmol) afforded, after RP-2 column chromatography, **34** as a purple oil (12.0 mg, 6% yield).

IR ν_max_ (ATR) 3374, 3069, 1699, 1684, 1499, 1205 cm^−1^; *R*_t_ = 9.53 min; ^1^H NMR (CD_3_OD, 400 MHz) δ_H_ 7.89 (1H, d, *J* = 5.8 Hz, H-2), 7.78 (1H, d, *J* = 8.0 Hz, H-4), 7.47 (2H, br s, H-6 and H-7), 7.14 (1H, s, H-10), 7.11–7.05 (2H, m, H-3 and H-5), 5.68 (1H, s, H-16), 3.15 (2H, t, *J* = 8.4 Hz, H_2_-13), 2.75 (2H, t, *J* = 7.2 Hz, H_2_-12), 2.22 (3H, s, H_3_-19), 1.87 (3H, s, H_3_-18); ^13^C NMR (CD_3_OD, 100 MHz) δ_C_ 170.9 (C-15), 153.4 (C-17), 151.0 (C-3a), 143.3 (C-2), 142.5 (C-7a), 137.9 (C-11), 136.1 (C-6), 130.6 (C-8a), 127.0 (C-11a), 126.0 (C-4), 123.8 (C-5), 122.7 (C-11b and C-10), 118.9 (C-16), 118.8 (C-7), 117.5 (C-9), 115.4 (C-3b), 105.4 (C-3), 39.2 (C-13), 32.5 (C-12), 27.5 (C-18), 20.3 (C-19); (+)-ESIMS *m/z* 360 [M + H]^+^; (+)-HRESIMS [M + H]^+^ 360.1723 (calcd. for C_22_H_22_N_3_O_2_, 360.1707).

##### 3.2.11.3. Styelsamine-*N*^14^-benzamide (**35**)

Using the general procedure, reaction of *N*-(3,4-dihydroxyphenethyl)benzamide (**30**) (43.0 mg, 0.17 mmol) with kynuramine dihydrobromide (57.0 mg, 0.18 mmol), CeCl_3_·7H_2_O (9.0 mg, 0.03 mmol) and Ag_2_O (97 mg, 0.42 mmol) afforded, after RP-2 column chromatography, **35** as a purple oil (13.0 mg, 15% yield).

IR ν_max_ (ATR) 3327, 3052, 1654, 1582, 1205 cm^−1^; *R*_t_ = 9.17 min; ^1^H NMR (CD_3_OD, 400 MHz) δ_H_ 11.66 (1H, s, NH-1), 8.97 (1H, t, *J* = 5.4 Hz, NH-14), 8.05 (1H, d, *J* = 8.0 Hz, H-2), 8.00 (1H, d, *J* = 7.8 Hz, H-4), 7.88 (2H, d, *J* = 7.6 Hz, H-17 and H-21), 7.70 (1H, d, *J* = 8.0 Hz, H-7), 7.63 (1H, t, *J* = 8.0 Hz, H-6), 7.56 (1H, dt, *J* = 6.4, 0.8 Hz, H-19), 7.47 (2H, t, *J* = 8.0 Hz, H-18 and H-20), 7.35–7.31 (2H, m, H-3 and H-10), 7.19 (1H, t, *J* = 7.8 Hz, H-5), 3.49 (2H, t, *J* = 7.6 Hz, H_2_-13), 3.05 (2H, t, *J* = 7.6 Hz, H_2_-12); ^13^C NMR (CD_3_OD, 100 MHz) δ_C_ 171.3 (C-15), 151.4 (C-3a), 143.5 (C-2), 142.7 (C-7a), 138.1 (C-11), 136.4 (C-6), 134.9 (C-16), 133.1 (C-19), 130.2 (C-11b), 129.7 (C-18 and C-20), 128.4 (C-17 and C-21), 127.6 (C-11a), 126.2 (C-4), 124.0 (C-5), 122.8 (C-10), 122.0 (C-8a), 119.2 (C-7), 117.5 (C-9), 115.5 (C-3b), 105.7 (C-3), 39.9 (C-13), 32.2 (C-12); (+)-ESIMS *m/z* 382 [M + H]^+^; (+)-HRESIMS [M + H]^+^ 382.1538 (calcd. for C_24_H_20_N_3_O_2_, 382.1550).

##### 3.2.11.4. Styelsamine-*N*^14^-2-phenylacetamide (**36**)

Using the general procedure, reaction of *N*-(3,4-dihydroxyphenethyl)-2-phenylacetamide (**31**) (142 mg, 0.52 mmol) with kynuramine dihydrobromide (178 mg, 0.55 mmol), CeCl_3_·7H_2_O (28.0 mg, 0.08 mmol) and Ag_2_O (360 mg, 1.57 mmol) afforded, after RP-2 column chromatography, **36** as a purple oil (28.0 mg, 11% yield).

IR ν_max_ (ATR) 3283, 3068, 1661, 1583 cm^−1^; *R*_t_ = 8.25 min; ^1^H NMR (CD_3_OD, 400 MHz) δ_H_ 8.05 (1H, d, *J* = 6.8 Hz, H-2), 8.00 (1H, d, *J* = 8.4 Hz, H-4), 7.62 (1H, br d, *J* = 8.2 Hz, H-6), 7.57 (1H, d, *J* = 8.2 Hz, H-7), 7.35–7.33 (1H, m, H-3), 7.33 (1H, s, H-10), 7.30–7.28 (3H, m, H-5, H-19 and H-21), 7.25–7.17 (3H, m, H-18, H-20 and H-22), 3.56 (2H, s, H_2_-16), 3.35 (2H, t, *J* = 8.0 Hz, H_2_-13), 2.94 (2H, t, *J* = 7.6 Hz, H_2_-12); ^13^C NMR (CD_3_OD, 100 MHz) δ_C_ 175.4 (C-15), 151.4 (C-3a), 143.5 (C-2), 142.7 (C-7a), 138.1 (C-11), 136.7 (C-17), 136.3 (C-6), 130.2 (C-18 and C-22), 129.7 (C-8a, C-19 and C-21), 128.1 (C-11a), 127.5 (C-20), 126.2 (C-4), 124.0 (C-5), 122.8 (C-10), 122.0 (C-11b), 119.1 (C-7), 117.3 (C-9), 115.5 (C-3b), 105.7 (C-3), 43.8 (C-16), 39.5 (C-13), 31.7 (C-12); (+)-ESIMS *m/z* 396 [M + H]^+^; (+)-HRESIMS [M + H]^+^ 396.1701 (calcd. for C_25_H_22_N_3_O_2_, 396.1707).

##### 3.2.11.5. Styelsamine-*N*^14^-3-phenylpropanamide (**37**)

Using the general procedure, reaction of *N*-(3,4-dihydroxyphenethyl)-3-phenylpropanamide (**32**) (80.0 mg, 0.28 mmol) with kynuramine dihydrobromide (96.0 mg, 0.30 mmol), CeCl_3_·7H_2_O (15.0 mg, 0.04 mmol) and Ag_2_O (163 mg, 0.70 mmol) afforded, after RP-2 column chromatography, **37** as a purple oil (29.0 mg, 20% yield).

IR ν_max_ (ATR) 3321, 1661, 1584, 1202, 1130, 1015 cm^−1^; *R*_t_ = 10.60 min; ^1^H NMR (DMSO-*d*_6_, 400 MHz) δ_H_ 13.49 (1H, br s, NH-1), 11.48 (1H, br s, OH), 10.80 (1H, br s, NH-8), 8.46 (1H, t, *J* = 5.6 Hz, NH-14), 8.26 (1H, d, *J* = 6.4 Hz, H-2), 8.22 (1H, d, *J* = 8.0 Hz, H-4), 7.71 (2H, d, *J* = 4.0 Hz, H-6 and H-7), 7.55 (1H, d, *J* = 6.4 Hz, H-3), 7.45 (1H, s, H-10), 7.28-7.14 (6H, m, H-5, H-19, H-20, H-21, H-22 and H-23), 3.28 (2H, dt, *J* = 6.8, 5.6 Hz, H_2_-13), 2.96 (2H, t, *J* = 6.8 Hz, H_2_-12), 2.85 (2H, t, *J* = 7.6 Hz, H_2_-17), 2.45 (2H, t, *J* = 7.6 Hz, H_2_-16); ^13^C NMR (DMSO-*d*_6_, 100 MHz) δ_C_ 173.2 (C-15), 149.2 (C-3a), 143.4 (C-2), 141.1 (C-7a and C-18), 136.7 (C-11), 135.0 (C-6), 128.3 (C-11a), 128.4 (C-8a), 128.2 (C-19, C-20, C-22 and C-23), 126.0 (C-21), 125.5 (C-4), 122.4 (C-5), 121.7 (C-10), 120.4 (C-11b), 117.7 (C-7), 116.2 (C-9), 113.9 (C-3b), 105.0 (C-3), 37.7 (C-13), 36.8 (C-16), 31.1 (C-17), 30.3 (C-12); (+)-ESIMS *m/z* 410 [M + H]^+^; (+)-HRESIMS [M + H]^+^ 410.1875 (calcd. for C_26_H_24_N_3_O_2_, 410.1863).

##### 3.2.11.6. Styelsamine-*N*^14^-palmitamide (**38**)

Using the general procedure, reaction of *N*-(3,4-dihydroxyphenethyl)palmitamide (**33**) (150.0 mg, 0.38 mmol) with kynuramine dihydrobromide (122 mg, 0.38 mmol), CeCl_3_.7H_2_O (2.0 mg, 0.05 mmol) and Ag_2_O (210 mg, 0.89 mmol) afforded, after RP-18 column chromatography, **38** as a purple oil (40 mg, 16% yield).

IR ν_max_ (ATR) 3286, 3074, 2917, 1685, 1560, 1511, 1467, 1200 cm^−1^; ^1^H NMR (CD_3_OD, 400 MHz) δ_H_ 8.13 (2H, d, *J* = 6.4 Hz, H-2 and H-4), 7.74–7.67 (2H, m, H-6 and H-7), 7.47 (1H, d, *J* = 6.8 Hz, H-3), 7.42 (1H, s, H-10), 7.27 (1H, t, *J* = 7.4 Hz, H-5), 3.37 (2H, t, *J* = 7.6 Hz, H_2_-13), 3.06 (2H, t, *J* = 7.6 Hz, H_2_-12), 2.26–2.24 (2H, m, H_2_-16), 1.65 (2H, t, *J* = 7.2 Hz, H_2_-17), 1.28 (24H, br s, H_2_-18–H_2_-29), 0.87 (3H, *J* = 7.2 Hz, H_3_-30); ^13^C NMR (CD_3_OD, 100 MHz) δ_C_ 177.8 (C-15), 151.7 (C-3a), 143.7 (C-2), 142.9 (C-7a), 138.2 (C-11), 136.4 (C-6), 130.4 (C-8a), 127.8 (C-11a), 126.3 (C-4), 124.1 (C-5), 122.9 (C-10), 122.3 (C-11b), 119.2 (C-7), 117.6 (C-9), 115.7 (C-3b), 105.8 (C-3), 39.4 (C-13), 37.0 (C-16), 33.1 (C-28), 32.1 (C-12), 30.8 (C-18–C-27), 26.9 (C-17), 23.8 (C-29), 14.5 (C-30); (+)-ESIMS *m/z* 516 [M + H]^+^; (+)-HRESIMS [M + H]^+^ 516.3589 (calcd. for C_33_H_46_N_3_O_2_, 516.3585).

#### 3.2.12. General Procedure for the Preparation of *N*-Acyl Cystodytin Analogues *1*, *10*, *39–42*

To a stirring solution of styelsamine analogue (1 equiv.) in MeOH (1.0 mL) was added Ag_2_O (1.5 equiv.) followed by sat. NaHCO_3_ (3 mL) dropwise. The purple mixture turned to red/orange then to yellow/green. The mixture was filtered, H_2_O (1.0 mL) and EtOAc (5.0 mL) added and the organic phase separated and dried *in vacuo* to afford the product as a yellow oil or solid.

##### 3.2.12.1. Cystodytin J (**10**)

Using the general procedure, reaction of styelsamine B (**13**) (7.0 mg, 0.016 mmol) with Ag_2_O (5.0 mg, 0.022 mmol) afforded **10** as a yellow oil (4.0 mg, 79% yield) [10]. 

^1^H NMR (CDCl_3_, 400 MHz) δ_H_ 8.94 (1H, d, *J* = 5.3 Hz, H-2), 8.59 (1H, d, *J* = 8.1 Hz, H-4), 8.55 (1H, d, *J* = 5.3 Hz, H-3), 8.32 (1H, dd, *J* = 8.4, 1.1 Hz, H-7), 7.91 (1H, dt, *J* = 8.4, 1.4 Hz, H-6), 7.83 (1H, dt, *J* = 8.1, 1.1 Hz, H-5), 6.95 (1H, s, H-10), 3.78 (2H, dt, *J* = 6.4, 6.4 Hz, H_2_-13), 3.32 (2H, t, *J* = 6.4 Hz, H_2_-12), 1.59 (3H, s, H_3_-16); (+)-ESIMS *m/z* 318 [M + H]^+^; (+)-HRESIMS [M + H]^+^ 318.1230 (calcd. for C_19_H_16_N_3_O_2_, 318.1237). ^1^H NMR data agreed with literature [4]. 

##### 3.2.12.2. Cystodytin A (**1**)

Using the general procedure, reaction of styelsamine analogue **34** (15.0 mg, 0.032 mmol) with Ag_2_O (8.0 mg, 0.042 mmol) afforded **1** as a yellow oil (7.0 mg, 62% yield).

^1^H NMR (CDCl_3_, 400 MHz) δ_H_ 9.24 (1H, d, *J* = 5.6 Hz, H-2), 8.60 (1H, dd, *J* = 7.8, 1.4 Hz, H-4), 8.57 (2H, d, *J* = 5.6 Hz, H-3), 8.32 (1H, dd, *J* = 7.8, 1.0 Hz, H-7), 7.94 (1H, dt, *J* = 7.8, 1.4 Hz, H-6), 7.85 (1H, dt, *J* = 7.8, 1.0 Hz, H-5), 6.95 (1H, s, H-10), 5.85 (1H, br s, NH-14), 5.50 (1H, s, H-16), 3.81 (2H, dt, *J* = 6.4, 6.4 Hz, H_2_-13), 3.36 (2H, t, *J* = 6.4 Hz, H_2_-12), 2.10 (3H, s, H_3_-19), 1.79 (3H, s, H_3_-18); (+)-ESIMS *m/z* 358 [M + H]^+^; (+)-HRESIMS [M + H]^+^ 358.1544 (calcd. for C_22_H_20_N_3_O_2_, 358.1550). ^1^H NMR data agreed with literature [2].

##### 3.2.12.3. Cystodytin-*N*^14^-benzamide (**39**)

Using the general procedure, reaction of styelsamine analogue **35** (20.0 mg, 0.040 mmol) with Ag_2_O (12.0 mg, 0.053 mmol) afforded **39** as a yellow oil (8.00 mg, 52% yield).

*R*_f_ (5% MeOH/CH_2_Cl_2_) 0.37; IR ν_max_ (smear) 3298, 1653, 1550, 1432, 1202, 766 cm^−1^; ^1^H NMR (CDCl_3_, 400 MHz) δ_H_ 9.24 (1H, d, *J* = 5.6 Hz, H-2), 8.60 (1H, d, *J* = 7.6 Hz, H-4), 8.58 (1H, d, *J* = 5.6 Hz, H-3), 8.30 (1H, d, *J* = 8.2 Hz, H-7), 7.93 (1H, dt, *J* = 8.2, 1.2 Hz, H-6), 7.86 (1H, t, *J* = 7.6 Hz, H-5), 7.65 (2H, d, *J* = 7.7 Hz, H-17 and H-21), 7.42 (1H, t, *J* = 1.1 Hz, H-19), 7.31 (2H, t, *J* = 7.7 Hz, H-18 and H-20), 7.01 (1H, s, H-10), 6.86 (1H, br s, NH-14), 3.98 (2H, dt, *J* = 6.4, 6.4 Hz, H_2_-13), 3.44 (2H, t, *J* = 6.4 Hz, H_2_-12); ^13^C NMR (CDCl_3_, 100 MHz) δ_C_ 181.8 (C-11), 173.8 (C-15), 152.7 (C-9), 151.6 (C-8a), 149.7 (C-2), 147.1 (C-11a), 145.3 (C-7a), 137.5 (C-3a), 134.4 (C-16), 132.1 (C-10), 131.9 (C-7), 131.3 (C-6), 131.2 (C-18 and C-20), 129.8 (C-5), 128.5 (C-19), 126.5 (C-17 and C-21), 123.0 (C-4), 121.4 (C-3b), 119.3 (C-3), 118.4 (C-11b), 40.0 (C-13), 31.0 (C-12); (+)-ESIMS *m/z* 380 [M + H]^+^; (+)-HRESIMS [M + H]^+^ 380.1411 (calcd. for C_24_H_18_N_3_O_2_ 380.1394).

##### 3.2.12.4. Cystodytin-*N*^14^-2-phenylacetamide (**40**)

Using the general procedure, reaction of styelsamine analogue **36** (21.0 mg, 0.041 mmol) with Ag_2_O (12.0 mg, 0.053 mmol) afforded **40** as a yellow oil (2.1 mg, 13% yield).

*R*_f_ (5% MeOH/CH_2_Cl_2_) 0.25; IR ν_max_ (smear) 3290, 2924, 1657, 1585, 1432, 1201, 722 cm^−1^; ^1^H NMR (CDCl_3_, 400 MHz) δ_H_ 9.23 (1H, d, *J* = 5.3 Hz, H-2), 8.58 (1H, d, *J* = 7.8 Hz, H-4), 8.55 (1H, d, *J* = 6.6 Hz, H-3), 8.25 (1H, d, *J* = 7.9 Hz, H-7), 7.92 (1H, t, *J* = 7.9 Hz, H-6), 7.84 (1H, t, *J* = 6.6 Hz, H-5), 7.21–7.18 (2H, m, H-19 and H-21), 7.16–7.11 (3H, m, H-18, H-20 and H-22), 6.81 (1H, s, H-10), 5.74 (1H, s, NH-14), 3.78–3.72 (2H, m, H_2_-13), 3.52 (2H, s, H_2_-16), 3.25 (2H, t, *J* = 6.3 Hz, H_2_-12); ^13^C NMR (CDCl_3_, 100 MHz) δ_C_ 183.4 (C-11), 171.2 (C-15), 151.8 (C-9), 150.3 (C-8a), 150.0 (C-2), 147.2 (C-11a), 145.4 (C-7a), 137.8 (C-3a), 134.6 (C-17), 132.8 (C-10), 132.0 (C-7), 131.7 (C-6), 129.9 (C-5), 129.3 (C-19 and C-21), 129.0 (C-18 and C-22), 127.3 (C-20), 122.9 (C-4), 121.9 (C-3b), 119.3 (C-3), 118.5 (C-11b), 43.8 (C-16), 39.3 (C-13), 31.3 (C-12); (+)-ESIMS *m/z* 394 [M + H]^+^; (+)-HRESIMS [M + H]^+^ 394.1552 (calcd. for C_25_H_20_N_3_O_2_ 394.1550).

##### 3.2.12.5. Cystodytin-*N*^14^-3-phenylpropanamide (**41**)

Using the general procedure, reaction of styelsamine analogue **37** (9.0 mg, 0.017 mmol) with Ag_2_O (5.0 mg, 0.022 mmol) afforded **41** as a yellow oil (5.0 mg, 71% yield).

*R*_f_ (5% MeOH/CH_2_Cl_2_) 0.13; IR ν_max_ (smear) 3285, 3072, 2922, 1647, 1551, 1588, 1332 cm^−1^; ^1^H NMR (CDCl_3_, 400 MHz) δ_H_ 9.20 (1H, d, *J* = 5.5 Hz, H-2), 8.59 (1H, dd, *J* = 7.6, 1.4 Hz, H-4), 8.51 (1H, d, *J* = 5.5 Hz, H-3), 8.28 (1H, dd, *J* = 7.6, 1.1 Hz, H-7), 7.94 (1H, dt, *J* = 7.6, 1.4 Hz, H-6), 7.85 (1H, t, *J* = 7.6, 1.1 Hz, H-5), 7.13 (2H, td, *J* = 7.5, 1.5 Hz, H-20 and H-22), 7.09–7.07 (2H, m, H-19 and H-23), 7.03 (1H, tt, *J* = 7.1, 1.4 Hz, H-21), 6.86 (1H, s, H-10), 6.05 (1H, br s, NH-14), 3.75 (2H, dt, *J* = 6.2, 6.2, H_2_-13), 3.24 (2H, t, *J* = 6.2 Hz, H_2_-12), 2.89 (2H, t, *J* = 7.6 Hz, H_2_-17), 2.44 (2H, t, *J* = 7.6 Hz, H_2_-16); ^13^C NMR (CDCl_3_, 100 MHz) δ_C_ 184.0 (C-11), 172.4 (C-15), 152.2 (C-9), 150.7 (C-8a), 150.2 (C-2), 147.0 (C-11a), 145.5 (C-7a), 140.9 (C-18), 137.4 (C-3a), 133.0 (C-10), 132.0 (C-6 and C-7), 130.1 (C-5), 128.5 (C-20 and C-22), 128.4 (C-19 and C-23), 126.3 (C-21), 123.1 (C-4), 122.1 (C-3b), 119.4 (C-3), 118.3 (C-11b), 39.5 (C-13), 38.7 (C-16), 31.9 (C-17), 31.6 (C-12); (+)-ESIMS *m/z* 407 [M + H]^+^; (+)-HRESIMS [M + H]^+^ 408.1716 (calcd. for C_26_H_22_N_3_O_2_, 408.1707).

##### 3.2.12.6. Cystodytin-*N*^14^-palmitamide (**42**)

Using the general procedure, reaction of styelsamine analogue **38** (37.0 mg, 0.059 mmol) with Ag_2_O (17.0 mg, 0.072 mmol) afforded **42** as a yellow oil (5.2 mg, 17% yield).

*R*_f_ (5% MeOH/CH_2_Cl_2_) 0.31; IR ν_max_ (smear) 3303, 2914, 2849, 1656, 1553, 1470, 774 cm^−1^; ^1^H NMR (CDCl_3_, 400 MHz) δ_H_ 9.25 (1H, d, *J* = 5.5 Hz, H-2), 8.61 (1H, dd, *J* = 8.1, 1.4 Hz, H-4), 8.58 (1H, d, *J* = 5.5 Hz, H-3), 8.32 (1H, dd, *J* = 7.5, 1.3 Hz, H-7), 7.95 (1H, td, *J* = 7.5, 1.4 Hz, H-6), 7.84 (1H, td, *J* = 8.1, 1.3 Hz, H-5), 6.95 (1H, s, H-10), 6.01 (1H, s, NH-14), 3.79 (2H, dt, *J* = 6.4, 6.4 Hz, H_2_-13), 3.32 (2H, t, *J* = 6.4 Hz, H_2_-12), 2.11 (2H, d, *J* = 7.4 Hz, H_2_-16), 1.55 (2H, br s, H_2_-17), 1.25 (24H, br s, H_2_-18–H_2_-29), 0.88 (3H, t, *J* = 7.1 Hz, H_3_-30); ^13^C NMR (CDCl_3_, 100 MHz) δ_C_ 184.7 (C-11), 173.4 (C-15), 151.9 (C-9), 150.6 (C-8a), 150.2 (C-2), 146.9 (C-11a), 145.5 (C-7a), 137.5 (C-3a), 133.0 (C-10), 132.0 (C-6 and C-7), 130.1 (C-5), 123.3 (C-4), 122.0 (C-3b), 119.5 (C-3), 117.2 (C-11b), 39.5 (C-13), 37.0 (C-16), 32.1 (C-28), 31.8 (C-12), 29.6 (C-18–C-27), 25.9 (C-17), 22.8 (C-29), 14.3 (C-30); (+)-ESIMS *m/z* 514 [M + H]^+^; (+)-HRESIMS [M + H]^+^ 514.3410 (calcd. for C_33_H_44_N_3_O_2_ 514.3428).

#### 3.2.13. Styelsamine D Ditrifluoroacetate (**15**)

Styelsamine B (**13**) (35.1 mg, 0.081 mmol) was dissolved in 1:1 MeOH/4N HCl (10 mL) and heated to 80 °C. After 24 h, the solvents were removed *in vacuo* and the product purified by RP-18 column chromatography (H_2_O (0.05% TFA):MeOH (0.05% TFA) (100:0 to 85:15)) to afford **15** as a purple oil (30.5 mg, 75% yield). 

IR ν_max_ (ATR) 3412, 1678, 1434, 1203, 1180, 1129, 765 cm^−1^; *R*_t_ = 4.68 min; ^1^H NMR (DMSO-*d*_6_, 400 MHz) δ_H_ 13.85 (1H, s, NH-1), 10.93 (1H, br s, OH), 8.32 (1H, d, *J* = 6.8 Hz, H-2), 8.25 (1H, d, *J* = 7.4 Hz, H-4), 8.03 (3H, br s, NH_3_-15), 7.75–7.68 (2H, m, H-6 and H-7), 7.62 (1H, d, *J* = 6.8 Hz, H-3), 7.51 (1H, s, H-10), 7.25 (1H, dt, *J* = 7.4, 1.5 Hz, H-5), 3.23 (2H, br t, *J* = 7.5 Hz, H_2_-12), 3.12–3.11 (2H, m, H_2_-13); ^13^C NMR (DMSO-*d*_6_, 100 MHz) δ_C_ 149.5 (C-3a), 143.6 (C-2), 141.5 (C-7a), 137.4 (C-11), 134.9 (C-6), 128.6 (C-8a), 126.8 (C-11a), 125.5 (C-4), 122.2 (C-5), 121.3 (C-10), 120.8 (C-11b), 118.1 (C-7), 114.2 (C-3b), 113.3 (C-9), 105.4 (C-3), 38.2 (C-13), 28.6 (C-12); (+)-ESIMS *m/z* 278 [M + H]^+^; (+)-HRESIMS [M + H]^+^ 278.1298 (calcd. for C_17_H_16_N_3_O, 278.1288). ^1^H and ^13^C NMR data agreed with literature [6].

#### 3.2.14. *O*-Methyl Styelsamine D Ditrifluoroacetate (**43**)

Styelsamine B (**13**) (19.0 mg, 0.044 mmol) was dissolved in 1:1 MeOH/4N HCl (10 mL) and heated to 80 °C. After 48 h, solvents were removed *in vacuo* and the residue purified by RP-18 column chromatography to afford **15** as a purple oil (13.4 mg, 60% yield) and **43** as a purple oil (10.3 mg, 45% yield). 

IR ν_max_ (ATR) 3382, 1675, 1429, 1178, 1138, 765 cm^−1^; *R*_t_ = 4.90 min; ^1^H NMR (DMSO-*d*_6_, 400 MHz) δ_H_ 13.85 (1H, br s, NH-1), 11.37 (1H, br s, NH-8), 8.38 (1H, d, *J* = 6.5 Hz, H-2), 8.28 (1H, d, *J* = 8.0 Hz, H-4), 8.18 (3H, br s, NH_3_-14), 8.04 (1H, d, *J* = 8.3 Hz, H-7), 7.79 (1H, s, H-10), 7.73–7.70 (2H, m, H-3 and H-6), 7.27 (1H, t, *J* = 8.0 Hz, H-5), 4.06 (3H, s, OMe), 3.38 (2H, t, *J* = 7.2 Hz, H_2_-12), 3.14–3.12 (2H, m, H_2_-13); ^13^C NMR (DMSO-*d*_6_, 100 MHz) δ_C_ 149.1 (C-3a), 143.5 (C-2), 141.2 (C-7a), 138.7 (C-11), 135.0 (C-6), 129.6 (C-8a), 127.3 (C-11a), 125.5 (C-4), 122.7 (C-5), 120.1 (C-11b), 119.5 (C-10), 118.0 (C-7), 117.9 (C-9), 113.8 (C-3b), 106.0 (C-3), 56.9 (OMe), 37.9 (C-13), 28.4 (C-12); (+)-ESIMS *m/z* 292 [M + H]^+^; (+)-HRESIMS [M + H]^+^ 292.1448 (calcd. for C_18_H_18_N_3_O 292.1444).

#### 3.2.15. *O*-Methyl-styelsamine-*N*^14^-3-methylbut-2-enamide Trifluoroacetate (**46**)

To a solution of *O*-methyl styelsamine D (**43**) (11.0 mg, 0.021 mmol) in dry DMF (3 mL) was added 3,3-dimethylacrylic acid (5.96 mg, 0.060 mmol) and PyBOP (31.0 mg, 0.060 mmol) followed by Et_3_N (22 µL, 0.16 mmol). The solution was stirred under N_2_ at room temperature for 25 h. CH_2_Cl_2_ (20 mL) was added, washed with H_2_O (15 mL) and the organic phase dried *in vacuo* to give a purple/red oil. Purification using RP-2 column chromatography (H_2_O (0.05% TFA):MeOH (0.05% TFA) (100:0 to 40:60)) afforded **46 **as a purple oil (9.1 mg, 88% yield). 

IR ν_max_ (ATR) 3396, 1675, 1586, 1433, 1182, 1134, 765 cm^−1^; *R*_t_ = 7.58 min; ^1^H NMR (DMSO-*d*_6_, 400 MHz) δ_H_ 13.78 (1H, br s, NH-1), 11.85 (1H, s, NH-8), 8.51 (1H, t, *J* = 5.6 Hz, NH-14), 8.34 (1H, d, *J* = 6.5 Hz, H-2), 8.28 (1H, d, *J* = 8.2 Hz, H-4), 7.79 (1H, d, *J* = 7.9 Hz, H-7), 7.76–7.72 (2H, m, H-6 and H-10), 7.68 (1H, d, *J* = 6.5 Hz, H-3), 7.27 (1H, td, *J* = 8.2, 1.0 Hz, H-5), 5.72 (1H, s, H-16), 4.03 (3H, s, OMe), 3.34 (2H, dt, *J* = 7.2, 5.6 Hz, H_2_-13), 3.08 (2H, t, *J* = 7.2 Hz, H_2_-12), 2.16 (3H, s, H_3_-19), 1.81 (3H, s, H_3_-18); ^13^C NMR (DMSO-*d*_6_, 100 MHz) δ_C_ 167.8 (C-15), 150.1 (C-17), 149.4 (C-3a), 143.5 (C-2), 141.2 (C-7a), 138.5 (C-11), 135.2 (C-6), 129.5 (C-8a), 126.7 (C-11a), 125.7 (C-4), 122.7 (C-5), 119.9 (C-11b), 119.1 (C-10), 118.3 (C-16), 118.1 (C-7), 117.7 (C-9), 114.1 (C-3b), 105.7 (C-3), 56.9 (OMe), 37.4 (C-13), 30.6 (C-12), 26.9 (C-18), 19.5 (C-19); (+)-ESIMS *m/z* 374 [M + H]^+^; (+)-HRESIMS [M + H]^+^ 374.1875 (calcd. for C_23_H_24_N_3_O_2_, 374.1876). ^1^H NMR data agreed with literature [2].

#### 3.2.16. *O*-Methyl-styelsamine-*N*^14^-2-phenylacetamide Trifluoroacetate (**47**)

To a solution of *O*-methyl styelsamine D (**43**) (11.0 mg, 0.021 mmol) in dry DMF (2 mL) was added phenylacetic acid (5.1 mg, 0.038 mmol) and PyBOP (19.0 mg, 0.038 mmol) followed by Et_3_N (16 µL, 0.11 mmol). The solution was stirred under N_2_ at room temperature for 1 h. CH_2_Cl_2_ (10 mL) was added, washed with H_2_O (10 mL) and the organic phase dried *in vacuo* to give a purple/red oil. Purification using RP-2 column chromatography (H_2_O (0.05% TFA):MeOH (0.05% TFA) (100:0 to 50:50)) afforded **47** as a purple oil (5.28 mg, 48% yield). 

IR ν_max_ (ATR) 3394, 1679, 1584, 1489, 1140, 1052, 708 cm^−1^; *R*_t_ = 7.33 min; ^1^H NMR (DMSO-*d*_6_, 400 MHz) δ_H_ 13.74 (1H, s, NH-1), 11.39 (1H, s, NH-8), 8.56 (1H, t, *J* = 5.6 Hz, NH-14), 8.35 (1H, d, *J* = 6.5 Hz, H-2), 8.27 (1H, d, *J* = 8.2 Hz, H-4), 7.76–7.74 (1H, m, H-7), 7.73–7.71 (2H, m, H-6 and H-10), 7.68 (1H, d, *J* = 6.5 Hz, H-3), 7.29–7.26 (1H, m, H-5), 7.29–7.23 (2H, m, H-18 and H-22), 7.23–7.19 (3H, m , H-19, H-20 and H-21), 4.00 (3H, s, OMe), 3.46 (2H, s, H_2_-16), 3.40 (2H, dt, *J* = 6.9, 5.6 Hz, H_2_-13), 3.08 (2H, t, *J* = 6.9 Hz, H_2_-12); ^13^C NMR (DMSO-*d*_6_, 100 MHz) δ_C_ 171.6 (C-15), 149.2 (C-3a), 143.5 (C-2), 141.0 (C-7a), 138.5 (C-11), 136.0 (C-17), 135.2 (C-6), 129.4 (C-8a), 128.8 (C-18 and C-22), 128.2 (C-19 and C-21), 126.7 (C-11a), 126.4 (C-20), 125.6 (C-4), 122.7 (C-5), 119.9 (C-11b), 119.0 (C-10), 118.2 (C-7), 116.0 (C-9), 114.0 (C-3b), 105.7 (C-3), 56.9 (OMe), 42.2 (C-16), 37.6 (C-13), 30.1 (C-12); (+)-ESIMS *m/z* 410 [M + H]^+^; (+)-HRESIMS [M + H]^+^ 410.1855 (calcd. for C_26_H_24_N_3_O_2_, 410.1863).

#### 3.2.17. *O*-Methyl-styelsamine-*N*^14^-3-phenylpropanamide Trifluoroacetate (**48**)

To a cold (0 °C) solution of *O*-methyl styelsamine D (**43**) (7.0 mg, 0.013 mmol) in dry THF (2 mL) was added dihydrocinnamoyl chloride (5.64 µL, 0.038 mmol) followed by Et_3_N (7.1 µL, 0.051 mmol). The solution was stirred under N_2_ at room temperature for 30 min. Solvents were removed *in vacuo* to give a purple/red oil. Purification using RP-2 (H_2_O (0.05% TFA):MeOH (0.05% TFA) (100:0 to 50:50)) and LH-20 column chromatography (MeOH (0.05% TFA)) afforded **48** as a purple oil (3.2 mg, 43% yield). 

IR ν_max_ (ATR) 3407, 1679, 1584, 1489, 1140, 1052, 701 cm^−1^; *R*_t_ = 7.85 min; ^1^H NMR (DMSO-*d*_6_, 400 MHz) δ_H_ 13.69 (1H, s, NH-1), 11.63 (1H, s, NH-8), 8.49 (1H, t, *J* = 5.5 Hz, NH-14), 8.36 (1H, d, *J* = 6.4 Hz, H-2), 8.31 (1H, d, *J* = 8.3 Hz, H-4), 7.80–7.74 (2H, m, H-6 and H-7), 7.76 (1H, s, H-10), 7.70 (1H, d, *J* = 6.4 Hz, H-3), 7.29 (1H, dt, *J* = 8.3, 2.2 Hz, H-5), 7.24 (2H, d, *J* = 7.4 Hz, H-19 and H-23), 7.21–7.15 (3H, m, H-20, H-21 and 22), 4.04 (3H, s, OMe), 3.33 (2H, dt, *J* = 7.2, 5.5 Hz, H_2_-13), 3.03 (2H, t, *J* = 7.2 Hz, H_2_-12), 2.86 (2H, t, *J* = 7.7 Hz, H_2_-17), 2.47–2.45 (2H, m, H_2_-16); ^13^C NMR (DMSO-*d*_6_, 100 MHz) δ_C_ 173.3 (C-15), 149.2 (C-3a), 143.5 (C-2), 141.0 (C-7a and C-18), 138.4 (C-11), 135.3 (C-6), 129.5 (C-8a), 128.2 (C-19, C-20, C-22 and C-23), 125.9 (C-11a and C-21), 125.7 (C-4), 122.7 (C-5), 119.9 (C-11b), 119.1 (C-10), 117.8 (C-7), 116.0 (C-9), 114.1 (C-3b), 105.7 (C-3), 56.9 (OMe), 37.5 (C-13), 36.8 (C-16), 31.0 (C-17), 30.3 (C-12); (+)-ESIMS *m/z* 424 [M + H]^+^; (+)-HRESIMS [M + H]^+^ 424.2005 (calcd. for C_27_H_26_N_3_O_2_, 424.2020).

### 3.3. Biological Assays

#### 3.3.1. Ethidium Bromide Displacement Assay

##### 3.3.1.1. Preparation of Solutions

An acetate buffer solution (pH 5) was prepared daily using NaOAc (2 mM), NaCl (9.3 mM) and Na_2_EDTA (0.1 mM). The acetate buffer was preferred in this displacement assay as the compounds being tested are easily oxidized (de-protonated); an instant color change from purple to yellow was observed in a pH 7 SHE buffer solution (NaCl [9.4 mM], EDTA [20µM] and HEPES [2 nM]) while a color change occur only after one and a half hours in acetate buffer solution. A stock solution of ethidium bromide (1.26 mM) was prepared by dissolving ethidium bromide (0.5 mg) in 1 mL of acetate buffer. A volume of 3 µL is required to make up a 3 mL DNA-ethidium bromide solution to give 1.26 µM of ethidium bromide.

The stock solutions of calf thymus DNA (CT-DNA) was prepared in an acetate buffer daily and then diluted down until absorbance of the CT-DNA at 260 nm is less than 1 absorbance unit. The concentration of CT-DNA solution was calculated in base pairs using the formula *A* = *εcl* (absorbance, *A*; absorption coefficient, *ε*, 13200 in base pairs [or 6600 in nucleotides] for CT-DNA; concentration, *c*, moles per liter; path length, *l*, 1 cm) [16]. Experiments that used a stock solution of CT-DNA that was older than four days were found to have lower initial fluorescence intensity, possibly due to the CT-DNA becoming partially hydrolyzed or depurinated, requiring the CT-DNA solution to be prepared daily. Once the concentration of the stock solution of CT-DNA was determined, the appropriate volume needed to make up a 3 mL DNA-ethidium bromide solution to give a total concentration of 1 µM of CT-DNA was then calculated.

The test compounds were prepared to a concentration of 1 mM using acetate buffer. In some cases (as indicated in Table 1) DMSO (up to 0.5% total volume) was also used if required to dissolve certain compounds. 

##### 3.3.1.2. General Methods for Competitive Ethidium Bromide Displacement Assay

All UV and fluorescence measurements were performed in 3 mL quartz cuvettes. Absorbance of CT-DNA solutions were measured using either a UV-2101 PC UV-VIS scanning Shimadzu Spectrophotometer or a Perkin-Elmer Lambda 35 UV/VIS spectrometer (at 260 nm). Fluorescence intensity for the ethidium bromide displacement assays were measured using a Perkin-Elmer LS 55 Luminescence Spectrometer (emission at 546 nm; excitation at 595 nm; emission slits at 10; excitation slits at 5). All solution used were at room temperature and stored in a freezer when they were not required. 

All glassware was washed with deionized H_2_O and dried with N_2_ gas before each experiment. Six DNA-ethidium bromide solutions were prepared by dilution with the acetate buffer to contain 1 µM CT-DNA and 1.26 µM ethidium bromide to make up a 3 mL solution. Each DNA-ethidium bromide solution was well mixed before measuring the initial florescence of the DNA-ethidium bromide solution in the cuvette. The fluorescence was reported as an average of five readings. Different volumes (constant differences, e.g., 3, 6, 9, 12, 15, 18 µL) of the test compound (1 mM) were added to each of the DNA-ethidium bromide test solutions. After equilibration for 15 min, the fluorescence of the solutions was measured in the order from lowest concentration to the highest concentration of test compound and reported from an average of five readings. Sets of volumes were screened until the fluorescence was below 50% of the initial reading. Once the volume was determined, the assay was repeated twice more to get an uncertainty value. Dilution effect was taken into consideration when the volume change is greater than 5% and fluorescence was corrected using the formula *F*_corr_ = *F*_exp_ × (3000 + V)/3000 [where V is the volume (in µL) of the compound added]. C_50_, concentration (µM) of compound required to reduce the fluorescence by 50%, was interpolated by graphing the concentration of the test compound versus the observed fluorescence. The apparent binding constants (*K*_app_) were calculated as follows: *K*_app_ = (1.26/C_50_) × *K*_ethidium_, with a value of *K*_ethidium_ = 2.1 × 10^6^ M(bp)^−1^ [16]. 

#### 3.3.2. Antitumor Testing

Details of the testing of compounds for antitumor activity under the auspices of the Developmental Therapeutics Program NCI/NIH are available elsewhere [27]. 

### 3.4. cLogP Calculations

The log P calculations were performed using the ALOGPS 2.1 software package [17,18]. 

## 4. Conclusions

A series of natural and un-natural analogues of the pyrido[4,3,2-*mn*]acridine styelsamine and pyridoacridone cystodytin marine natural products were prepared and evaluated for DNA binding affinity and whole cell antiproliferative properties against a panel of human tumor cell lines. Overall it was found that styelsamine analogues were stronger DNA binders, with the natural products styelsamines B and D having particularly high affinity. In comparison the cystodytin iminoquinone alkaloids showed lower affinity for DNA, but were just as active as styelsamine analogues at inhibiting proliferation of tumor cells *in vitro*. Whole cell activity of both styelsamines and cystodytins correlated with lipophilicity, with the most potent growth inhibition properties being associated with alkaloids from both series with clogP ~4.0–4.5. These results will direct future efforts to optimize the antiproliferative activity of this class of natural products.

## Supplementary Files

Supplementary File 1 Supplementary Information (PDF, 662 KB)

## References

[1] Molinski T.F. (1993). Marine pyridoacridine alkaloids: Structure, synthesis, and biological chemistry. Chem. Rev..

[2] Kobayashi J., Cheng J.-F., Wälchli M.R., Nakamura H., Hirata Y., Sasaki T., Ohizumi Y. (1988). Cystodytins A, B, and C, novel tetracyclic aromatic alkaloids with potent antineoplastic activity from the Okinawan tunicate *Cystodytes dellechiajei*. J. Org. Chem..

[3] Kobayashi J., Tsuda M., Tanabe A., Ishibashi M., Cheng J.-F., Yamamura S., Sasaki T. (1991). Cystodytins D-I, new cytotoxic tetracyclic aromatic alkaloids from the Okinawan marine tunicate *Cystodytes dellechiajei*. J. Nat. Prod..

[4] McDonald L.A., Eldredge G.S., Barrows L.R., Ireland C.M. (1994). Inhibition of topoisomerase II catalytic activity by pyridoacridine alkaloids from a *Cystodytes* sp. ascidian: A mechanism for the apparent intercalator-induced inhibition of topoisomerase II. J. Med. Chem..

[5] Appleton D.R., Pearce A.N., Lambert G., Babcock R.C., Copp B.R. (2002). Isodiplamine, cystodytin K and lissoclinidine: Novel bioactive alkaloids from the New Zealand ascidian *Lissoclinum notti*. Tetrahedron.

[6] Copp B.R., Jompa J., Tahir A., Ireland C.M. (1998). Styelsamines A–D: New tetracyclic pyridoacridine alkaloids from the Indonesian ascidian *Eusynstyela latericius*. J. Org. Chem..

[7] Skyler D., Heathcock C.H. (2002). The pyridoacridine family tree: A useful scheme for designing synthesis and predicting undiscovered natural products. J. Nat. Prod..

[8] Bry D., Banaigs B., Long C., Bontemps N. (2011). New pyridoacridine alkaloids from the purple morph of the ascidian *Cystodytes dellechiajei*. Tetrahedron Lett..

[9] Marshall K.M., Barrows L.R. (2004). Biological activities of pyridoacridines. Nat. Prod. Rep..

[10] Skyler D., Heathcock C.H. (2001). A simple biomimetic synthesis of styelsamine B. Org. Lett..

[11] Rudi A., Kashman Y. (1989). Six new alkaloids from the purple Red Sea tunicate *Eudistoma* sp. J. Org. Chem..

[12] Plubrukarn A., Davidson B.S. (1998). Arnoamines A and B, new cytotoxic pentacyclic pyridoacridine alkaloids from the ascidian *Cystodytes* sp. J. Org. Chem..

[13] Bouffier L., Baldeyrou B., Hildebrand M.-P., Lansiaux A., David-Cordonnier M.-H., Carrez D., Croisy A., Renaudet O., Dumya P., Demeunynck M. (2006). Amino- and glycoconjugates of pyrido[4,3,2-*kl*]acridine. Synthesis, anti-tumor activity, and DNA binding. Bioorg. Med. Chem..

[14] Bouffier L., Gosse I., Demeunynck M., Mailley P. (2012). Electrochemistry and bioactivity relationship of 6-substituted-4*H*-pyrido[4,3,2-*kl*]acridin-4-one antitumor drug candidates. Bioelectrochemistry.

[15] LePecq J.B., Paoletti C. (1967). A fluorescent complex between ethidium bromide and nucleic acids. J. Mol. Biol..

[16] Jenkins T.C., Fox K.R. (1997). Optical absorbance and fluorescence techniques for measuring DNA-drug interaction. Drug-DNA Interaction Protocols.

[17] Tetko I.V., Gasteiger J., Todeschini R., Mauri A., Livingstone D., Ertl P., Palyulin V.A., Radchenko E.V., Zefirov N.S., Makarenko A.S., Tanchuk V.Y., Prokopenko V.V. (2005). Virtual computational chemistry laboratory—design and description. J. Comput. Aid. Mol. Des..

[18] (2005). ALOGPS 2.1.

[19] Snell R.H., Woodward R.L., Willis M.C. (2011). Catalytic enantioselective total synthesis of hodgkinsine B. Angew. Chem. Int. Ed..

[20] Nakagawa M., Okajima H., Hino T. (1977). Photosensitized oxygenation of N_b_-methoxycarbonyltryptamines. A new pathway to kynurenine derivatives. J. Am. Chem. Soc..

[21] Weissbach H., Smith T.E., Daly J.W., Witkop B., Udenfriend S. (1960). A rapid spectrophotometric assay of monoamine oxidase based on the rate of disappearance of kynuramine. J. Biol. Chem..

[22] Johannes M., Altmann K.-H. (2012). A ring-closing metathesis-based approach to the synthesis of (+)-tetrabenazine. Org. Lett..

[23] Minor D.L., Wyrick S.D., Charifson P.S., Watts V.J., Nichols D.E., Mailman R.B. (1994). Synthesis and molecular modeling of 1-phenyl-1,2,3,4-tetrahydroisoquinolines and related 5,6,8,9-Tetrahydro-13b*H*-dibenzo[*a*,*h*]quinolizines as D_1_ dopamine antagonists. J. Med. Chem..

[24] Nagubandi S., Fodor G. (1980). The mechanism of the Bischler-Napieralski reaction. J. Heterocycl. Chem..

[25] Burke T.R., Fesen M.R., Mazumder A., Wang J., Carothers A.M., Grunberger D., Driscoll J., Kohn K., Pommier Y. (1995). Hydroxylated aromatic inhibitors of HIV-1 integrase. J. Med. Chem..

[26] Dang H.T., Kang G.J., Yoo E.S., Hong J., Choi J.S., Kim H.S., Chung H.Y., Jung J.H. (2011). Evaluation of endogenous fatty acid amides and their synthetic analogues as potential anti-inflammatory leads. Bioorg. Med. Chem..

[27] Developmental Therapeutics Program NCI/NIH. http://dtp.nci.nih.gov/.

