# Supplementary material for: Synthesis, DNA Binding and Antitumor Evaluation of Styelsamine and Cystodytin Analogues
Source: Mar Drugs. 2013 Jan 28;11(2):274–99. doi: 10.3390/md11020274 (PMC3640380; doi:10.3390/md11020274)
Supplement: Supplementary File 1 — Supplementary Information (PDF, 662 KB) [file marinedrugs-11-00274-s001.pdf]

Supplementary Information

**Figure S1.**	NCI one dose (10 μM) data for styelsamine B (**13**)	2
**Figure S2.**	NCI one dose (10 μM) data for styelsamine D (**15**)	3
**Figure S3.**	NCI one dose (10 μM) data for styelsamine analogue **34**	4
**Figure S4.**	NCI one dose (10 μM) data for styelsamine analogue **35**	5
**Figure S5.**	NCI one dose (10 μM) data for styelsamine analogue **37**	6
**Figure S6.**	NCI one dose (10 μM) data for styelsamine analogue **38**	7
**Figure S7.**	NCI one dose (10 μM) data for *O*-methyl styelsamine D (**43**)	8
**Figure S8.**	NCI one dose (10 μM) data for *O*-methyl styelsamine analogue **46**	9
**Figure S9.**	NCI one dose (10 μM) data for *O*-methyl styelsamine analogue **48**	10
**Figure S10.**	NCI one dose (10 μM) data for cystodytin A (**1**)	11
**Figure S11.**	NCI one dose (10 μM) data for cystodytin J (**10**)	12
**Figure S12.**	NCI one dose (10 μM) data for cystodytin analogue **39**	13
**Figure S13.**	NCI one dose (10 μM) data for cystodytin analogue **41**	14

Figure S1. NCI one dose (10 µM) data for styelsamine B (**13**).

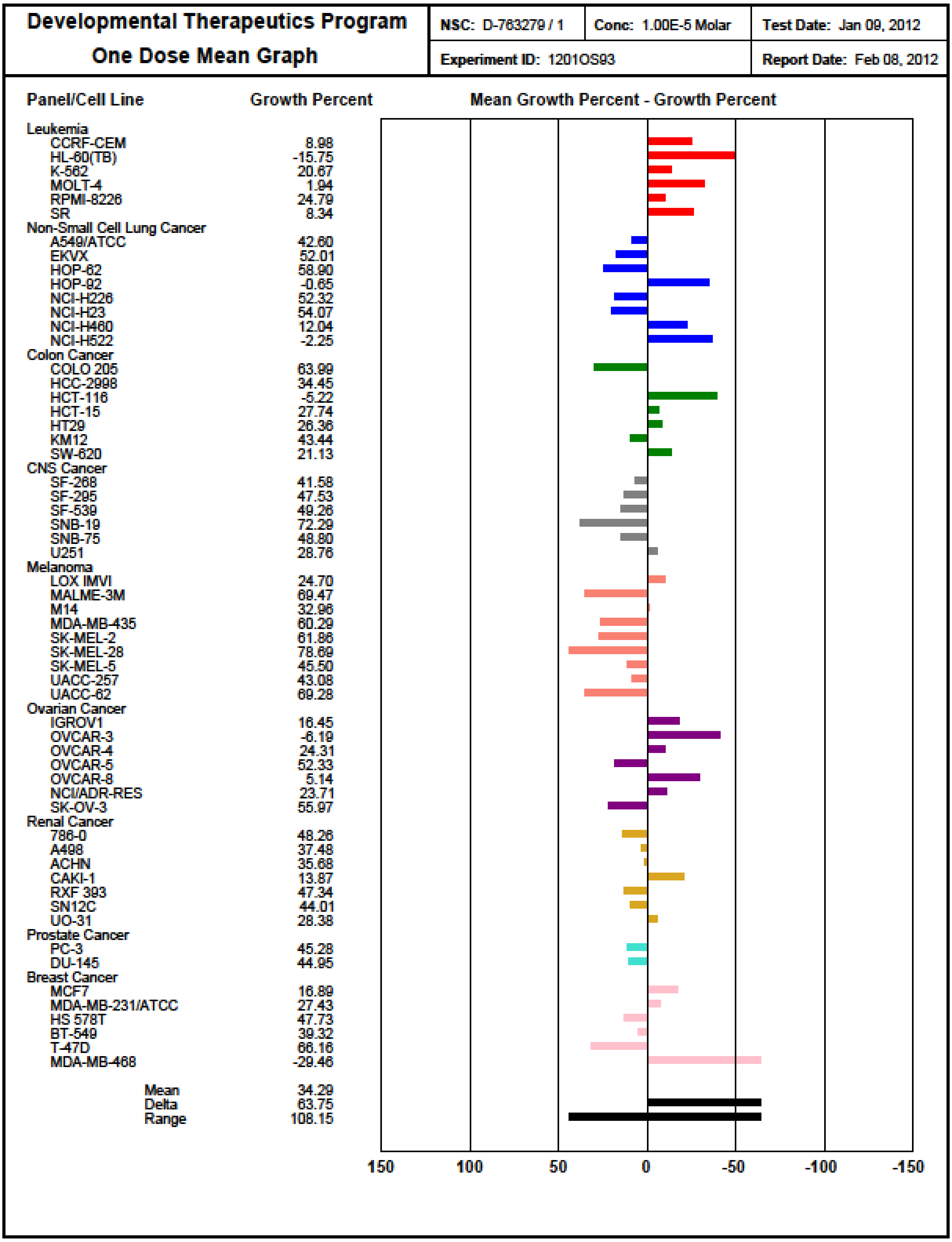

Figure S2. NCI one dose (10 µM) data for styelsamine D (**15**).

Developmental Therapeutics Program One Dose Mean Graph	NSC: D-763284 / 1	Conc: 1.00E-5 Molar	Test Date: Jan 09, 2012
	Experiment ID: 1201OS93		Report Date: Feb 08, 2012

Panel/Cell Line	Growth Percent
**Leukemia**	
HL-60(TB)	74.47
K-562	53.23
MOLT-4	58.05
RPMI-8226	63.03
SR	5.92
**Non-Small Cell Lung Cancer**	
A549/ATCC	25.14
EKVX	85.66
HOP-62	41.95
HOP-92	28.95
NCI-H226	7.74
NCI-H23	10.16
NCI-H460	2.49
NCI-H522	42.30
**Colon Cancer**	
COLO 205	79.45
HCC-2998	74.70
HCT-116	46.92
HCT-15	53.71
HT29	74.04
KM12	85.46
SW-620	36.62
**CNS Cancer**	
SF-268	56.85
SF-295	22.87
SF-539	20.05
SNB-19	27.68
SNB-75	21.06
U251	25.86
**Melanoma**	
LOX IMVI	37.88
MALME-3M	61.89
M14	13.48
MDA-MB-435	75.51
SK-MEL-2	94.98
SK-MEL-28	70.75
SK-MEL-5	41.50
UACC-257	49.55
UACC-62	39.54
**Ovarian Cancer**	
IGROV1	57.19
OVCAR-3	82.93
OVCAR-4	73.10
OVCAR-5	72.15
OVCAR-8	44.34
NCI/ADR-RES	54.80
SK-OV-3	91.75
**Renal Cancer**	
786-0	34.95
A498	83.95
ACHN	13.38
CAKI-1	19.56
RXF 393	50.55
SN12C	9.44
UO-31	28.79
**Prostate Cancer**	
PC-3	59.60
DU-145	31.08
**Breast Cancer**	
MCF7	37.93
MDA-MB-231/ATCC	81.67
HS 578T	82.89
BT-549	96.38
T-47D	57.30
MDA-MB-468	58.34
Mean	49.68
Delta	47.19
Range	93.89

Mean Growth Percent - Growth Percent

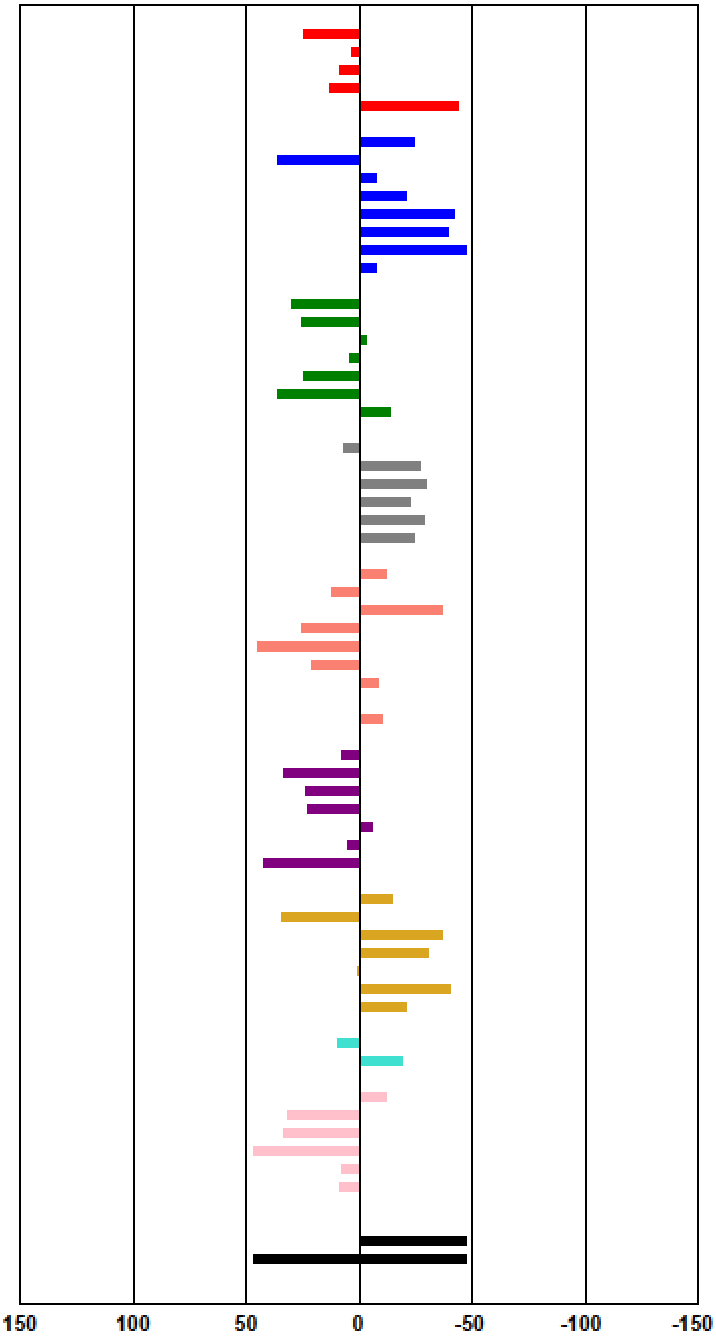

Figure S3. NCI one dose (10 µM) data for styelsamine analogue **34**.

Developmental Therapeutics Program
One Dose Mean Graph

NSC: D-763280 / 1	Conc: 1.00E-5 Molar	Test Date: Jan 09, 2012
Experiment ID: 1201OS93		Report Date: Feb 08, 2012

Panel/Cell Line	Growth Percent
Leukemia	
CCRF-CEM	15.22
HL-60(TB)	0.60
K-562	18.37
MOLT-4	19.72
RPMI-8226	29.42
SR	8.04
Non-Small Cell Lung Cancer	
A549/ATCC	50.04
EKVX	36.53
HOP-62	74.43
NCI-H226	17.79
NCI-H23	23.70
NCI-H460	10.21
NCI-H522	42.96
Colon Cancer	
COLO 205	102.21
HCC-2998	55.51
HCT-116	3.10
HCT-15	31.34
HT29	81.75
KM12	65.63
SW-620	33.64
CNS Cancer	
SF-268	39.61
SF-295	65.18
SF-539	35.94
SNB-19	60.30
SNB-75	55.53
U251	28.87
Melanoma	
LOX IMVI	30.60
MALME-3M	48.32
M14	4.58
MDA-MB-435	50.71
SK-MEL-2	38.65
SK-MEL-28	58.39
SK-MEL-5	36.00
UACC-257	30.85
UACC-62	36.67
Ovarian Cancer	
IGROV1	46.94
OVCAR-3	11.10
OVCAR-4	51.01
OVCAR-5	64.04
OVCAR-8	36.82
NCI/ADR-RES	41.60
SK-OV-3	84.97
Renal Cancer	
786-0	12.47
A498	10.33
ACHN	12.10
CAKI-1	1.76
RXF 393	24.69
SN12C	18.66
UO-31	7.10
Prostate Cancer	
PC-3	62.04
DU-145	36.53
Breast Cancer	
MCF7	39.41
MDA-MB-231/ATCC	29.44
HS 578T	24.50
BT-549	41.78
T-47D	74.87
MDA-MB-468	12.22
Mean	36.58
Delta	35.98
Range	101.61

Mean Growth Percent - Growth Percent

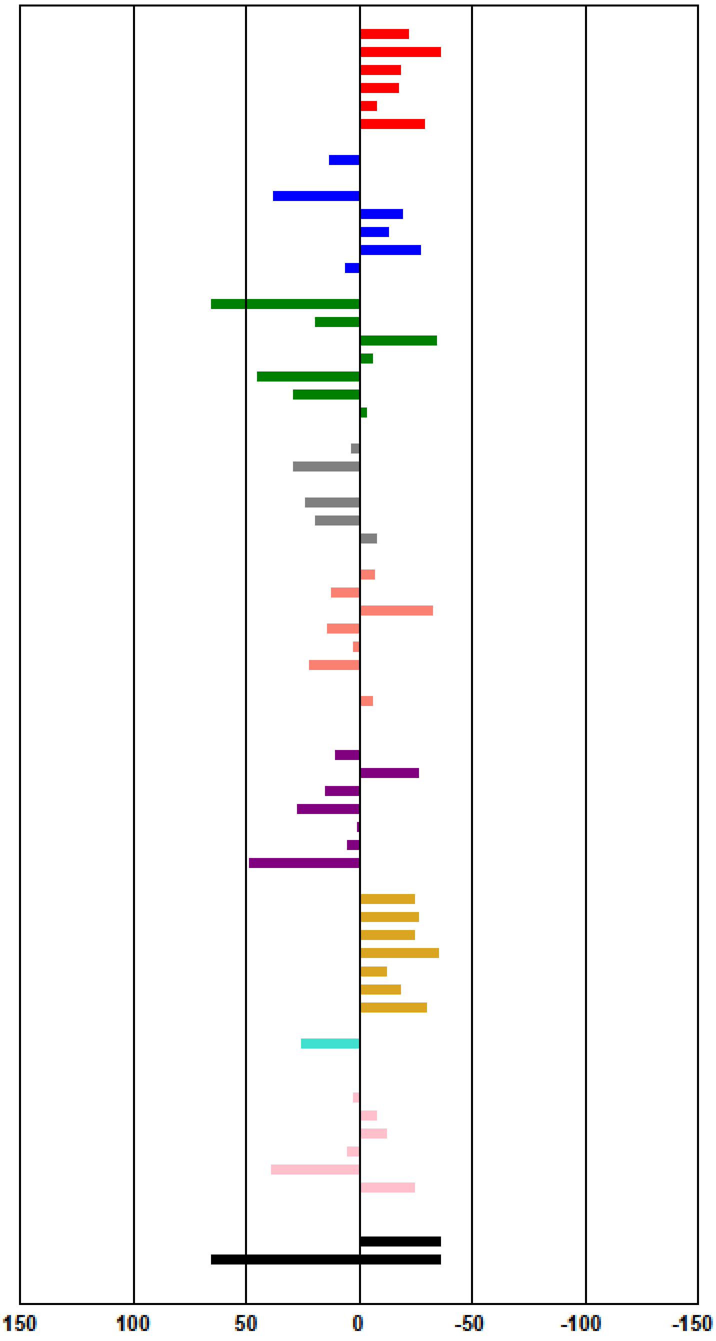

Figure S4. NCI one dose (10 µM) data for styelsamine analogue **35**.

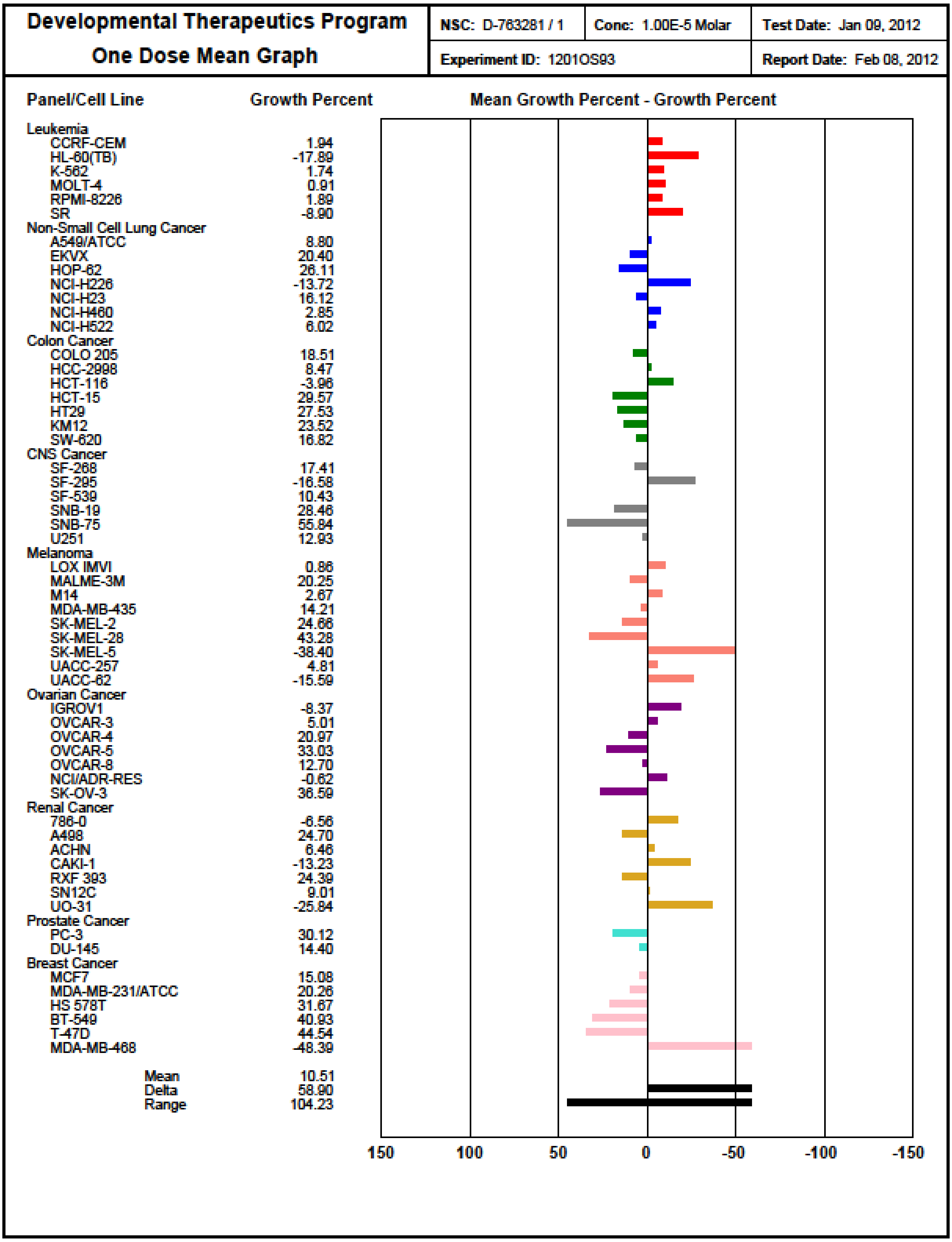

Figure S5. NCI one dose (10 µM) data for styelsamine analogue **37**.

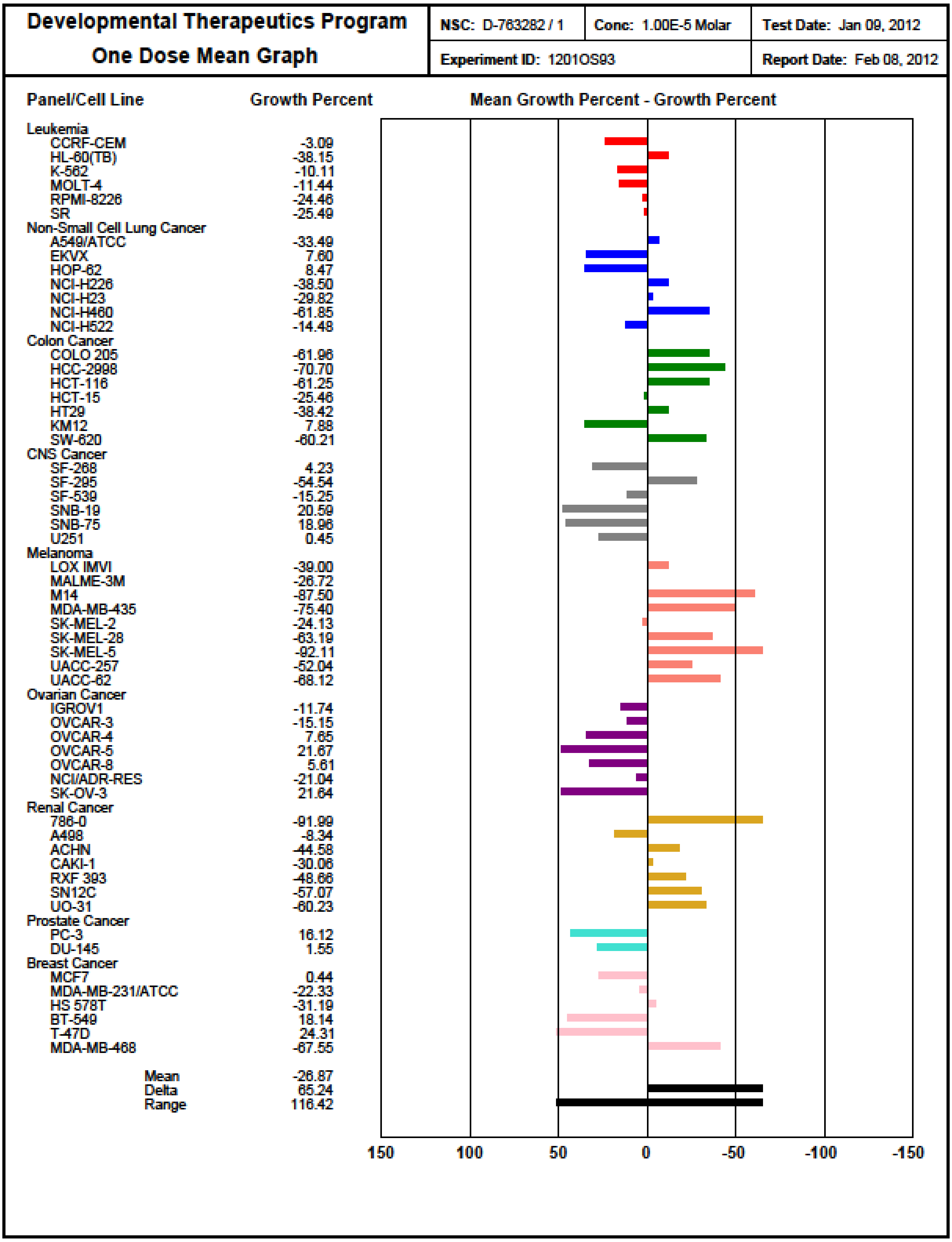

Figure S6. NCI one dose (10 µM) data for styelsamine analogue **38**.

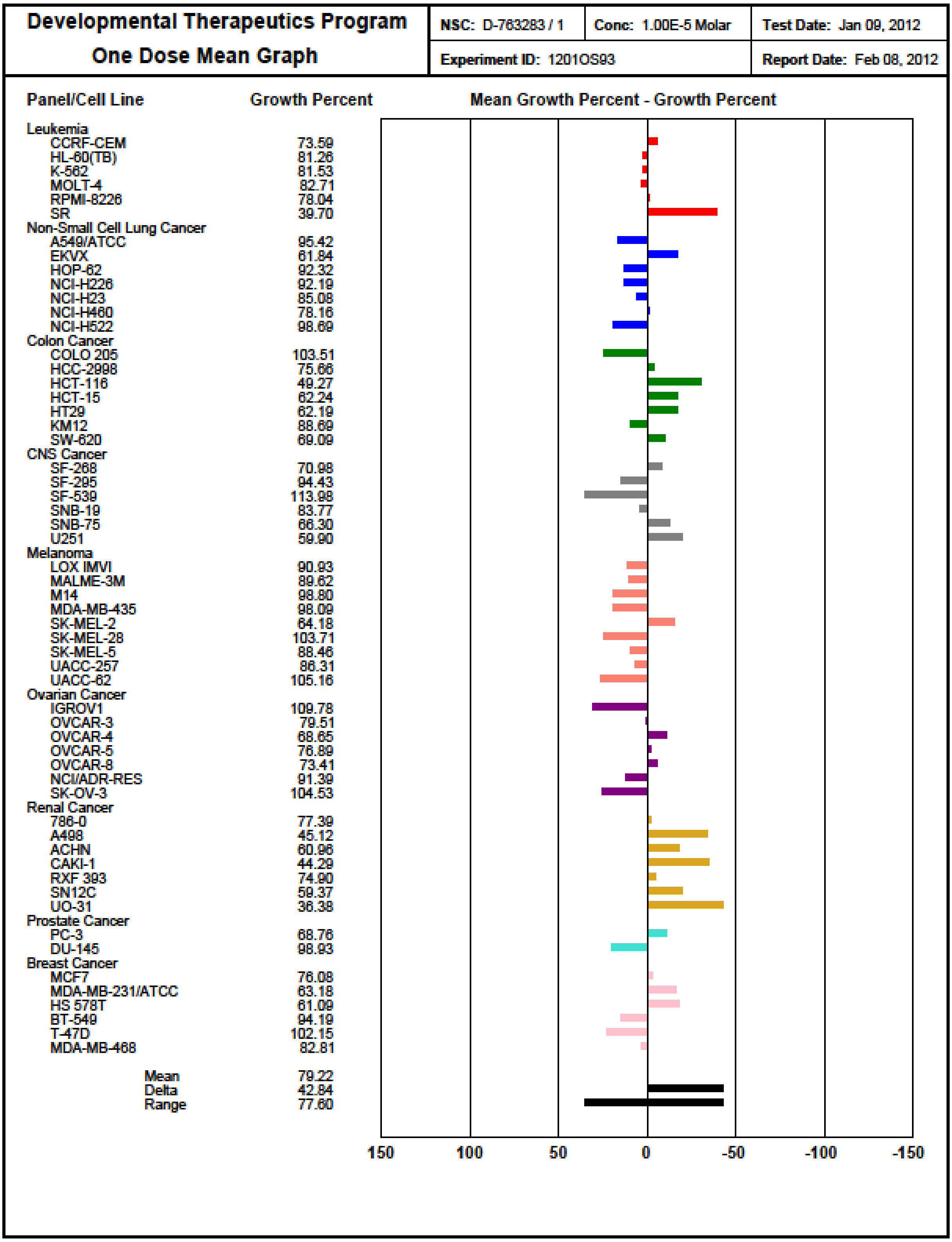

Figure S7. NCI one dose (10 μM) data for *O*-methyl styelsamine D (**43**).

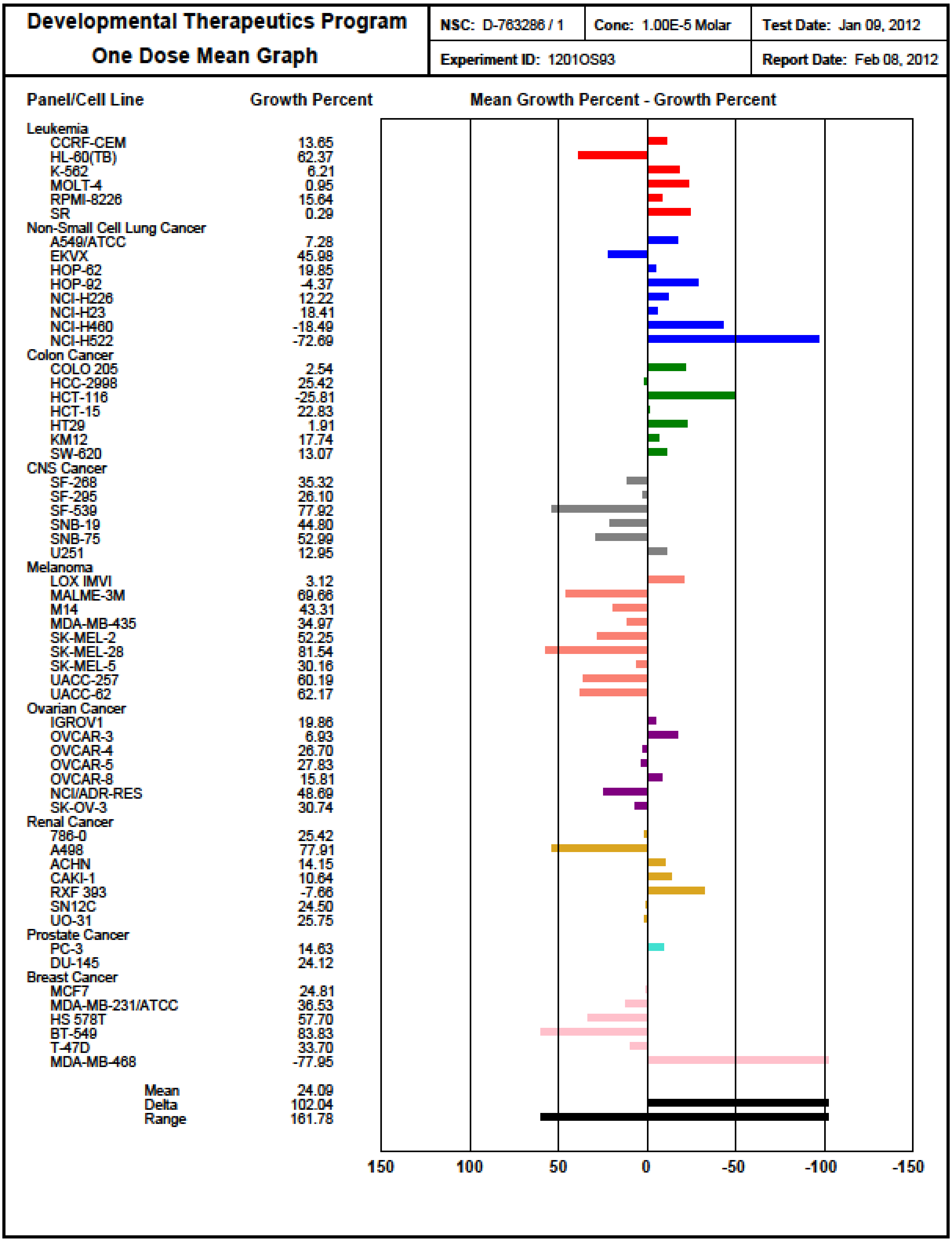

Figure S8. NCI one dose (10 µM) data for *O*-methyl styelsamine analogue **46**.

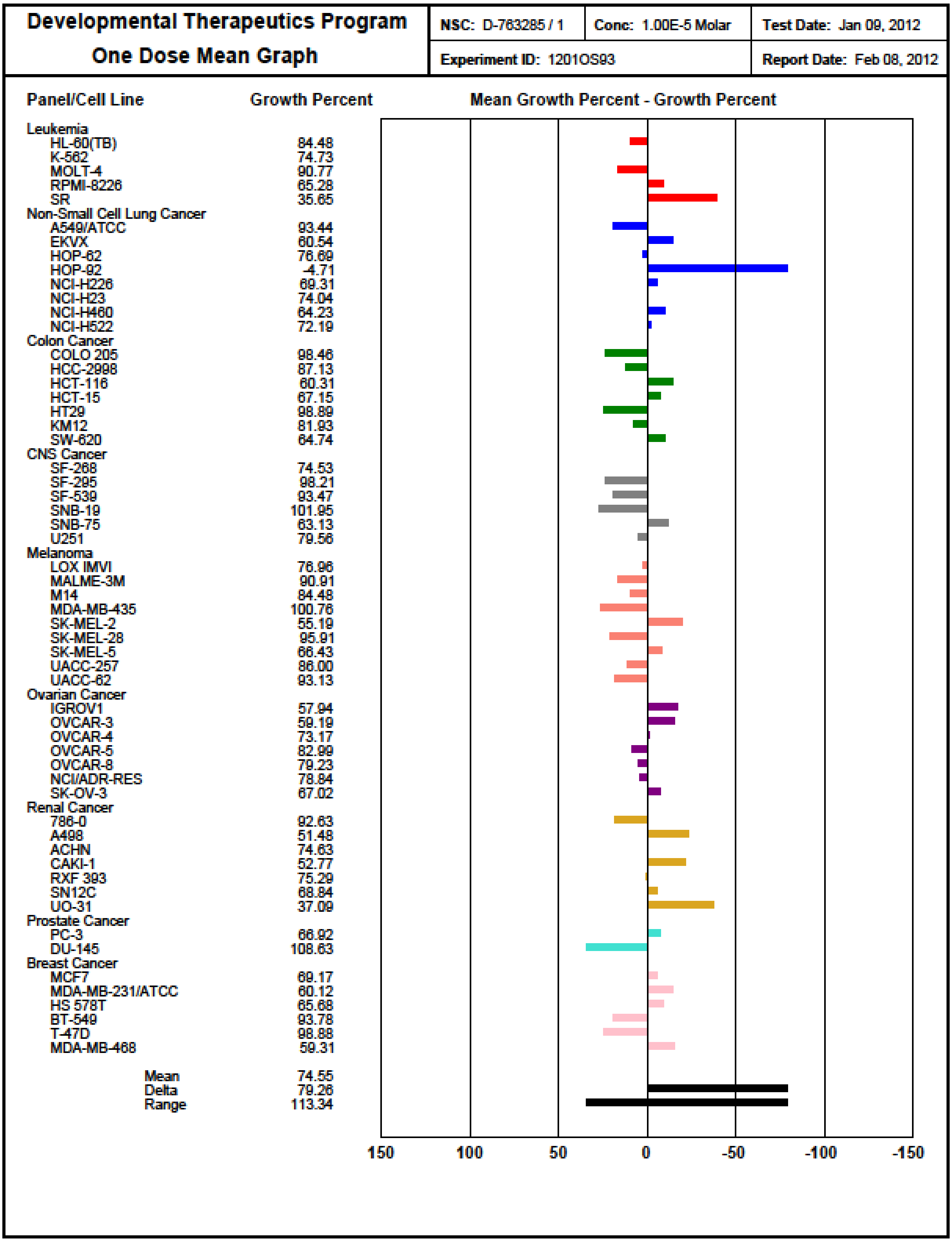

Figure S9. NCI one dose (10 µM) data for O-methyl styelsamine analogue **48**.

Developmental Therapeutics Program One Dose Mean Graph	NSC: D-763287 / 1	Conc: 1.00E-5 Molar	Test Date: Jan 09, 2012
	Experiment ID: 1201OS93		Report Date: Feb 08, 2012

Panel/Cell Line	Growth Percent
**Leukemia**	
CCRF-CEM	51.51
HL-60(TB)	28.82
K-562	32.52
MOLT-4	54.19
RPMI-8226	31.56
SR	6.40
**Non-Small Cell Lung Cancer**	
A549/ATCC	46.23
EKVX	43.01
HOP-62	62.40
HOP-92	-19.26
NCI-H226	54.57
NCI-H23	55.57
NCI-H460	22.70
NCI-H522	46.66
**Colon Cancer**	
COLO 205	68.23
HCC-2998	48.54
HCT-116	11.16
HCT-15	28.59
HT29	68.58
KM12	61.39
SW-620	42.77
**CNS Cancer**	
SF-268	51.79
SF-295	62.10
SF-539	66.73
SNB-19	69.04
SNB-75	33.96
U251	43.36
**Melanoma**	
LOX IMVI	35.53
MALME-3M	68.12
M14	47.96
MDA-MB-435	71.38
SK-MEL-2	43.54
SK-MEL-28	79.45
SK-MEL-5	49.79
UACC-257	68.37
UACC-62	71.19
**Ovarian Cancer**	
IGROV1	11.61
OVCAR-3	14.57
OVCAR-4	42.97
OVCAR-5	56.95
OVCAR-8	56.10
NCI/ADR-RES	55.49
SK-OV-3	53.31
**Renal Cancer**	
786-0	58.33
A498	9.22
ACHN	32.49
CAKI-1	10.02
RXF 393	62.08
SN12C	34.71
UO-31	23.72
**Prostate Cancer**	
PC-3	49.31
DU-145	55.48
**Breast Cancer**	
MCF7	49.87
MDA-MB-231/ATCC	43.17
HS 578T	34.14
BT-549	65.43
T-47D	88.72
MDA-MB-468	28.46
Mean	45.60
Delta	64.86
Range	107.98

Mean Growth Percent - Growth Percent

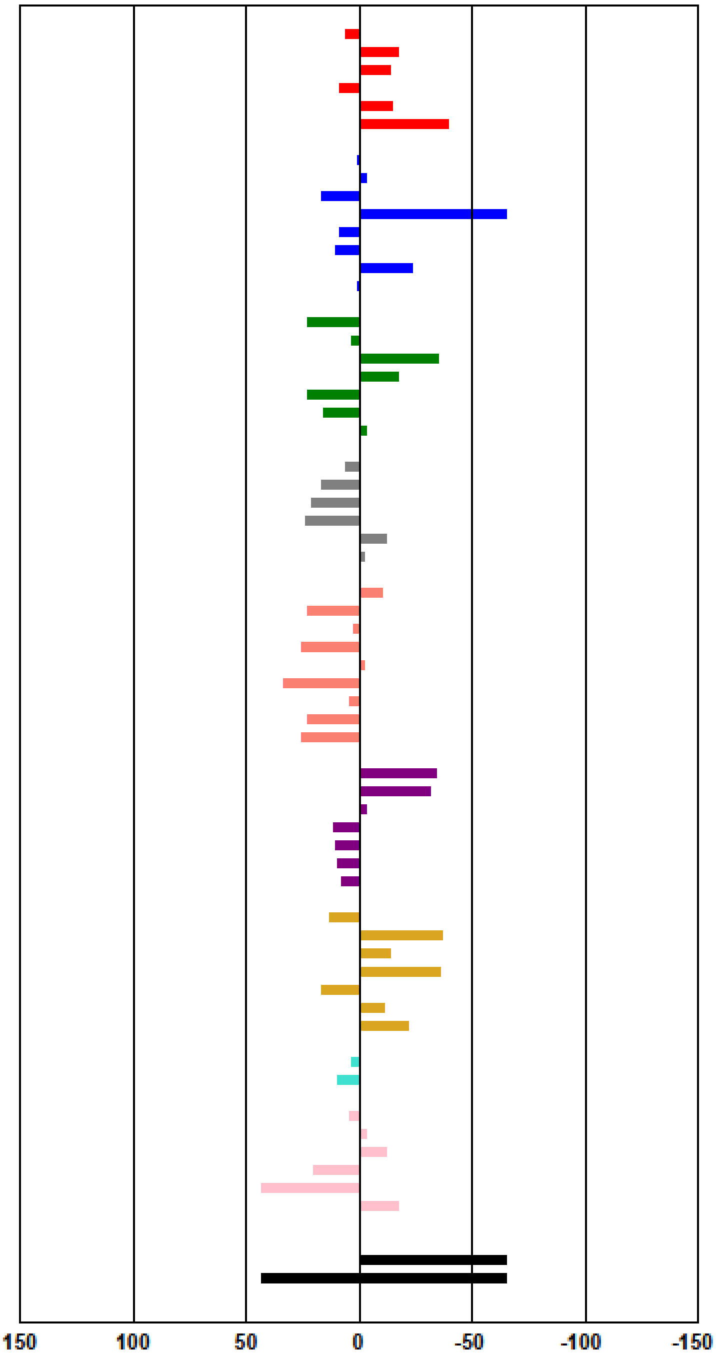

Figure S10. NCI one dose (10 µM) data for cystodytin A (**1**).

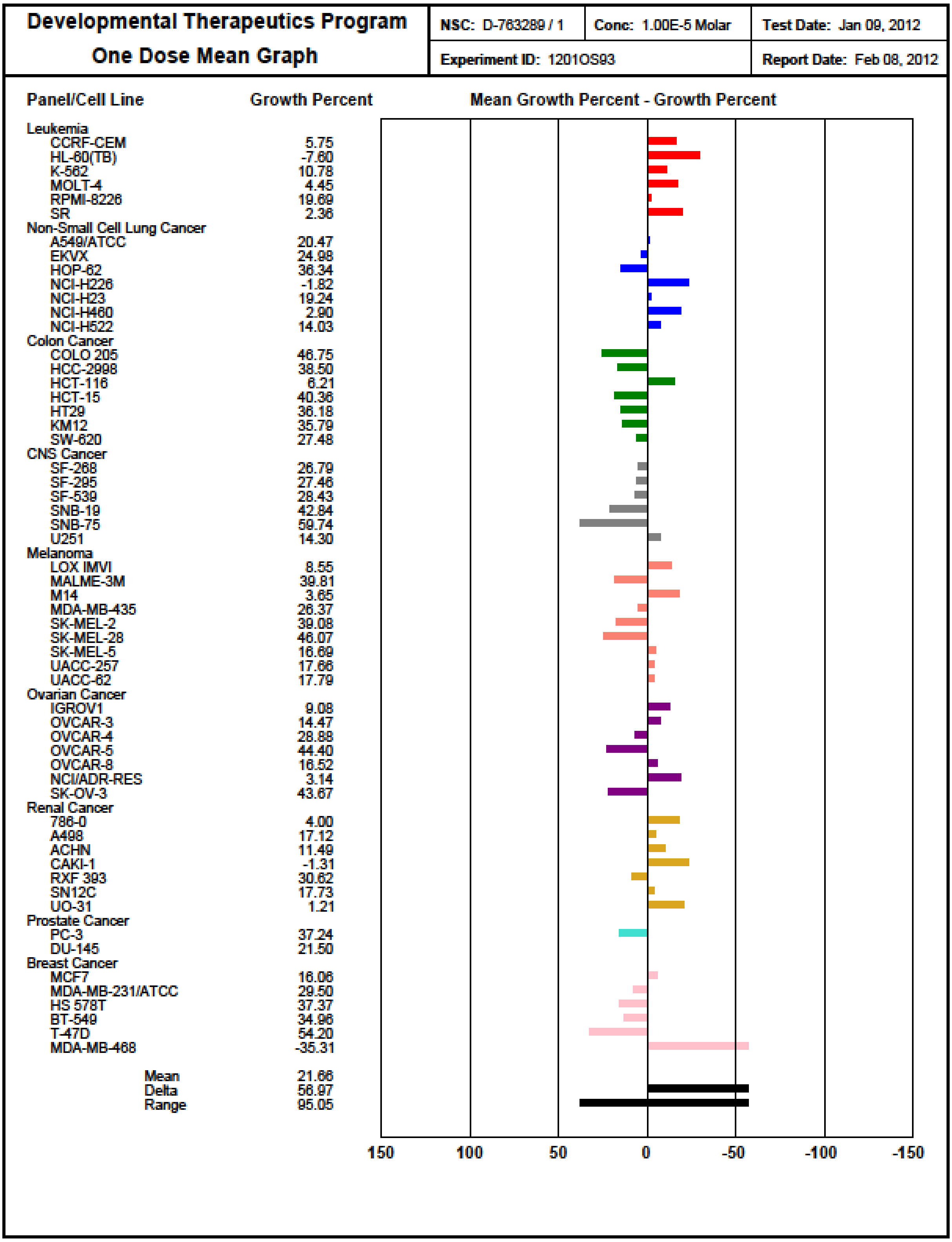

Figure S11. NCI one dose (10 µM) data for cystodytin J (**10**).

Developmental Therapeutics Program One Dose Mean Graph	NSC: D-763291 / 1	Conc: 1.00E-5 Molar	Test Date: Jan 09, 2012
	Experiment ID: 1201OS93		Report Date: Feb 08, 2012

Panel/Cell Line	Growth Percent
**Leukemia**	
CCRF-CEM	62.41
HL-60(TB)	84.34
K-562	23.94
MOLT-4	75.92
RPMI-8226	69.16
SR	10.13
**Non-Small Cell Lung Cancer**	
A549/ATCC	55.30
EKVX	82.13
HOP-62	87.38
NCI-H226	82.21
NCI-H23	52.88
NCI-H460	20.92
NCI-H522	75.25
**Colon Cancer**	
COLO 205	110.75
HCC-2998	75.70
HCT-116	39.87
HCT-15	84.77
HT29	103.47
KM12	93.95
SW-620	55.35
**CNS Cancer**	
SF-268	67.01
SF-295	69.20
SF-539	94.41
SNB-19	72.04
SNB-75	46.14
U251	50.00
**Melanoma**	
LOX IMVI	64.34
MALME-3M	83.38
M14	66.86
MDA-MB-435	85.30
SK-MEL-2	97.24
SK-MEL-28	90.28
SK-MEL-5	85.65
UACC-257	92.84
UACC-62	72.92
**Ovarian Cancer**	
IGROV1	86.38
OVCAR-3	77.63
OVCAR-4	80.18
OVCAR-5	106.90
OVCAR-8	64.21
NCI/ADR-RES	80.09
SK-OV-3	91.97
**Renal Cancer**	
786-0	87.76
A498	62.81
ACHN	23.46
CAKI-1	45.53
RXF 393	84.29
SN12C	49.92
UO-31	54.02
**Prostate Cancer**	
PC-3	69.09
DU-145	76.00
**Breast Cancer**	
MCF7	63.01
MDA-MB-231/ATCC	42.74
HS 578T	44.65
BT-549	83.29
T-47D	88.76
MDA-MB-468	97.00
Mean	70.93
Delta	60.80
Range	100.62

Mean Growth Percent - Growth Percent

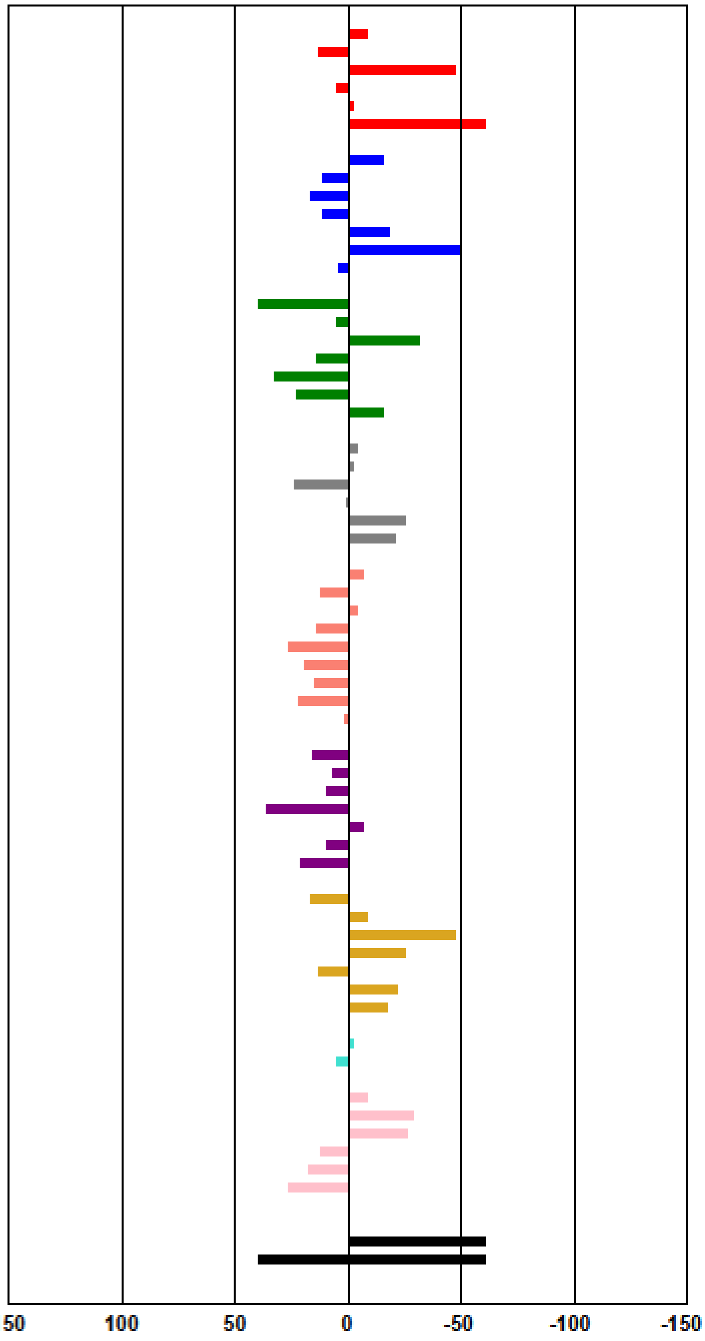

Figure S12. NCI one dose (10 µM) data for cystodytin analogue **39**.

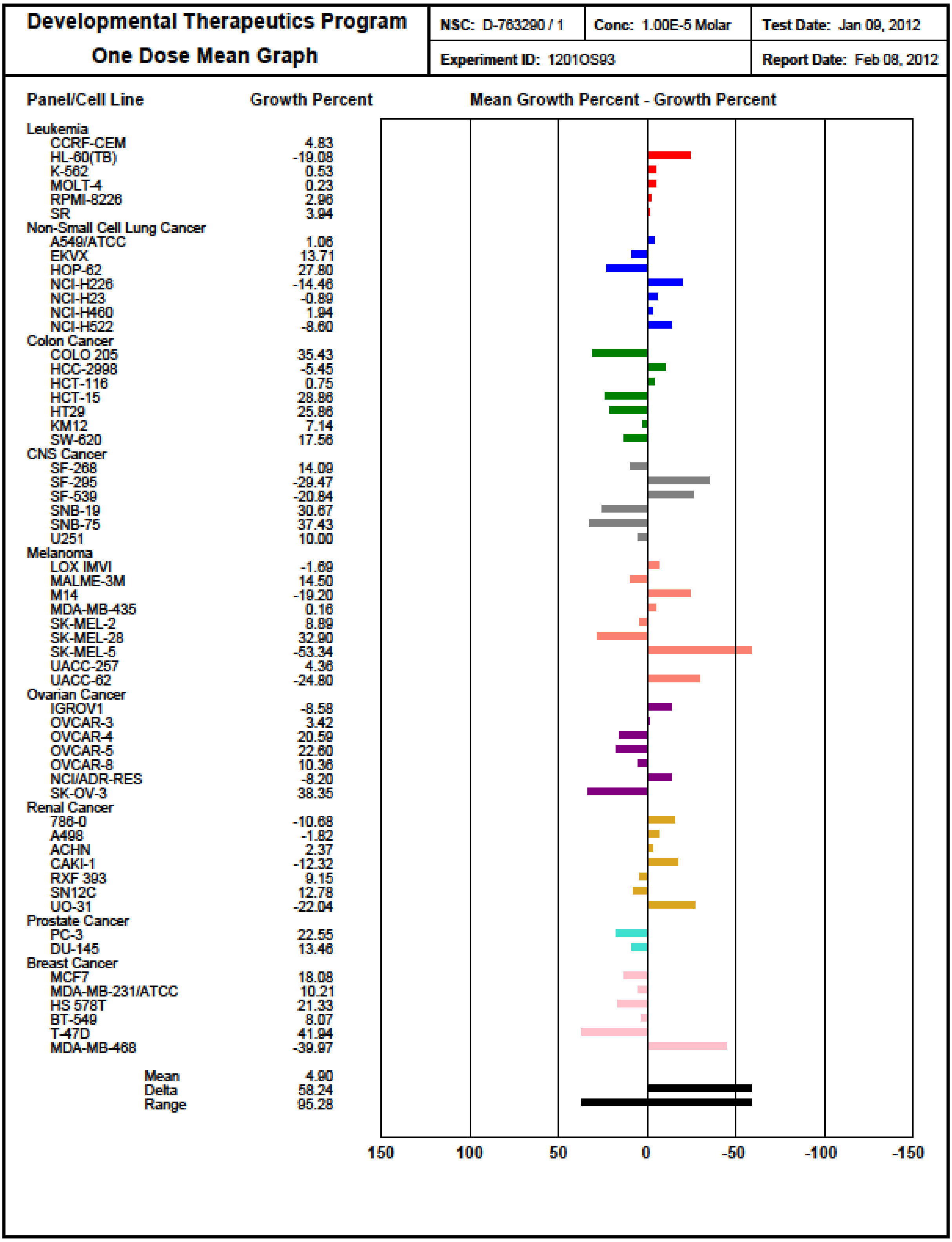
Developmental Therapeutics Program
One Dose Mean Graph

NSC: D-763290 / 1 | Conc: 1.00E-5 Molar | Test Date: Jan 09, 2012
Experiment ID: 1201OS93 | Report Date: Feb 08, 2012

Panel/Cell Line	Growth Percent
**Leukemia**	
CCRF-CEM	4.83
HL-60(TB)	-19.08
K-562	0.53
MOLT-4	0.23
RPMI-8226	2.96
SR	3.94
**Non-Small Cell Lung Cancer**	
A549/ATCC	1.06
EKVX	13.71
HOP-62	27.80
NCI-H226	-14.46
NCI-H23	-0.89
NCI-H460	1.94
NCI-H522	-8.60
**Colon Cancer**	
COLO 205	35.43
HCC-2998	-5.45
HCT-116	0.75
HCT-15	28.86
HT29	25.86
KM12	7.14
SW-620	17.56
**CNS Cancer**	
SF-268	14.09
SF-295	-29.47
SF-539	-20.84
SNB-19	30.67
SNB-75	37.43
U251	10.00
**Melanoma**	
LOX IMVI	-1.69
MALME-3M	14.50
M14	-19.20
MDA-MB-435	0.16
SK-MEL-2	8.89
SK-MEL-28	32.90
SK-MEL-5	-53.34
UACC-257	4.36
UACC-62	-24.80
**Ovarian Cancer**	
IGROV1	-8.58
OVCAR-3	3.42
OVCAR-4	20.59
OVCAR-5	22.60
OVCAR-8	10.36
NCI/ADR-RES	-8.20
SK-OV-3	38.35
**Renal Cancer**	
786-0	-10.68
A498	-1.82
ACHN	2.37
CAKI-1	-12.32
RXF 393	9.15
SN12C	12.78
UO-31	-22.04
**Prostate Cancer**	
PC-3	22.55
DU-145	13.46
**Breast Cancer**	
MCF7	18.08
MDA-MB-231/ATCC	10.21
HS 578T	21.33
BT-549	8.07
T-47D	41.94
MDA-MB-468	-39.97
Mean	4.90
Delta	58.24
Range	95.28

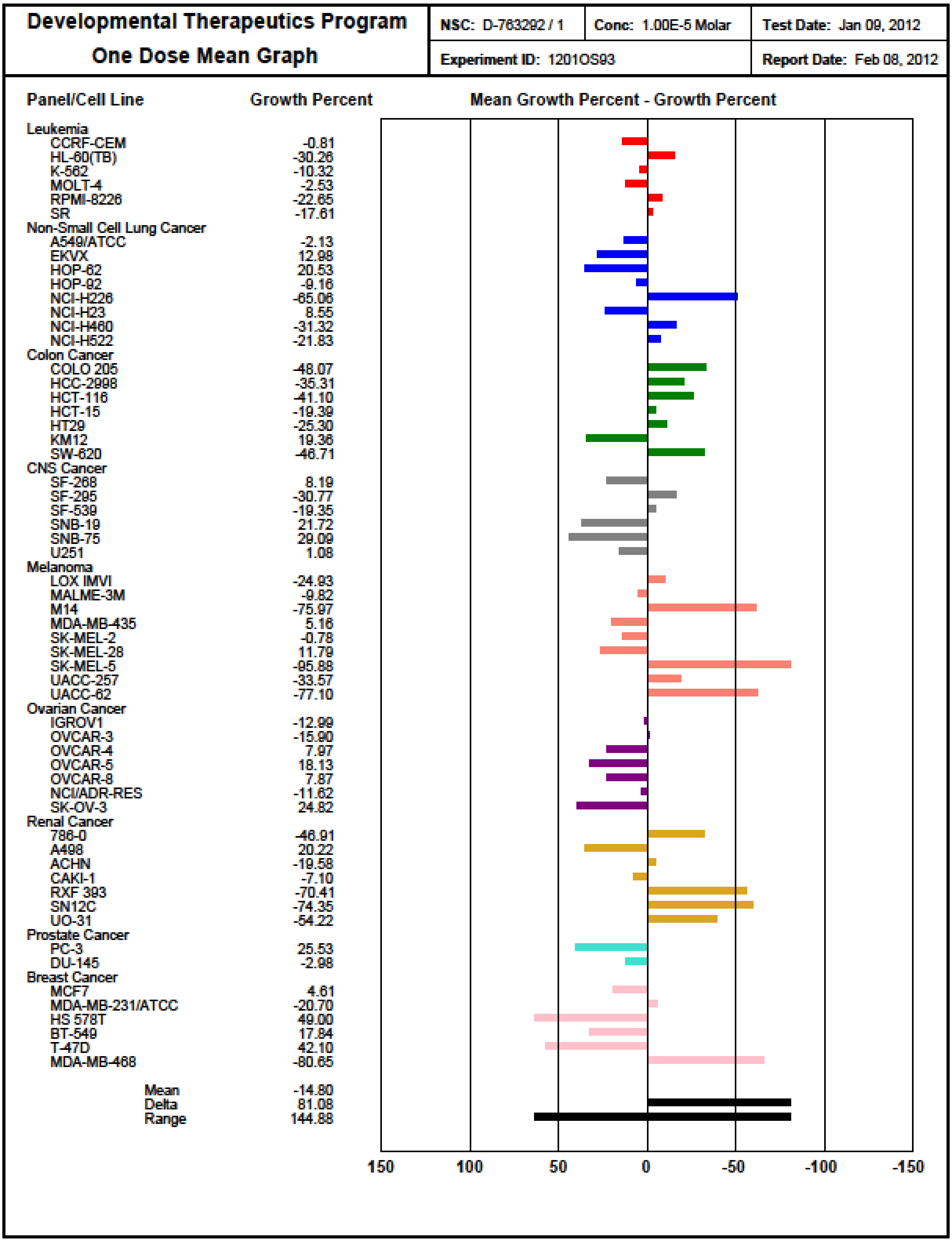

Figure S13. NCI one dose (10 µM) data for cystodytin analogue **41**.
